# A human NK cell progenitor that originates in the thymus and generates KIR^+^NKG2A^−^ NK cells

**DOI:** 10.1126/sciadv.adv9650

**Published:** 2025-08-08

**Authors:** Julian Reiß, Sujal Ghosh, Michael Scheid, Lea Graafen, Nadine Scherenschlich, Sandra Weinhold, Katharina Raba, Stefan Paulusch, Elena De Dominico, Thi X. U. Pham, Marc Beyer, Hans-Jürgen Laws, Tim Niehues, Arndt Borkhardt, Markus Uhrberg, Sabrina B. Bennstein

**Affiliations:** ^1^ Institute for Transplantation Diagnostics and Cell Therapeutics, Medical Faculty, Heinrich-Heine University Düsseldorf, Moorenstr. 5, Düsseldorf, Germany.; ^2^ Department of Pediatric Oncology, Hematology and Clinical Immunology, Medical Faculty, Center of Child and Adolescent Health, Heinrich-Heine-University, Düsseldorf, Germany.; ^3^ Herzzentrum Duisburg, Evangelisches Klinikum Niederrhein, Gerrickstraße 21, Duisburg, Germany.; ^4^ PRECISE Platform for Genomics and Epigenomics at German Center for Neurodegenerative Diseases (DZNE) and University of Bonn and West German Genome Center, Bonn, Germany.; ^5^ Department of Pediatrics, Helios Klinikum Krefeld, Krefeld, Germany.; ^6^ Immunogenomics & Neurodegeneration, German Center for Neurodegenerative Diseases (DZNE), Bonn, Germany.; ^7^ Institute of Immunology, Faculty of Medicine, RWTH Aachen University, Aachen, Germany.

## Abstract

KIR^+^NKG2A^−^ natural killer (NK) cells have the unique ability to detect down-regulation of single HLA-I allotypes, frequently occurring in malignantly transformed and virus-infected cells. We have recently shown that circulating innate lymphoid cells 1 (cILC1s) have the potential to generate such KIR^+^NKG2A^−^ NK cells, but their developmental origin was unknown. Here, we demonstrate that the development of cILC1 is thymus dependent and identify a putative progenitor of cILC1s in the thymus (thyILC1). Single-cell RNA sequencing analysis revealed a close relationship of thyILC1s to CD34^+^ double-negative thymocytes. Both generated comparable NK cell frequencies, while only thyILC1s could be efficiently differentiated into KIR^+^NKG2A^−^ NK cells. Last, patients with *FOXN1* haploinsufficiency, showing congenital thymic hypoplasia, exhibited a profound deficiency of cILC1s but not cILC2s and cILC3s, demonstrating their specific thymus dependency. Together, the data suggest that thyILC1s are the source of a thymus-dependent NK cell differentiation pathway that promotes generation of KIR^+^NKG2A^−^ NK cells.

## Teaser

A thymus-dependent NK cell differentiation pathway leads to the generation of KIR^+^NKG2A^−^ NK cells.

## INTRODUCTION

Innate lymphoid cells (ILCs) are important drivers of innate immunity and guardians of homeostasis as well as integrity of mucosal barriers (*1*). The three major ILC subsets have been described to mirror T cell functionality, where ILC1s, ILC2s, and ILC3s correspond to T helper 1 (T_H_1), T_H_2, and T_H_17 cells, respectively (*2*). While this concept is still valid for tissue-resident ILCs (tILCs), circulating ILCs (cILCs) are in many aspects distinct from their tILC counterparts (*3*). Whereas CD117^−/+^CRTH2^+^ cILC2s are indeed phenotypically and functionally closely related to tILC2 (*4*, *5*), recent studies revealed that cILC1s and cILC3s lack the established functionality and cytokine responsiveness of tILC1s and tILC3s but have progenitor potential and are able to differentiate into the full spectrum of innate lymphocytes (*6*, *7*). In this regard, we have previously shown that cILC1s from umbilical cord blood (CB) as well as peripheral blood (PB) can be efficiently differentiated into natural killer (NK) cells (*7*).

cILC1s are unique among human NK cell progenitors (NKPs) in their ability to efficiently differentiate in vitro into NK cells expressing killer-cell immunoglobulin-like receptors (KIRs) but lacking the lectin-like inhibitory receptor NKG2A (*7*). The lack of NKG2A expression makes these KIR^+^NKG2A^−^ (“KIR-only”) NK cells resistant to inhibition via human leukocyte antigen E (HLA-E), a nonclassical HLA-I molecule that is maintained or even overexpressed on many different kinds of tumors (*8*). NKG2A is widely expressed on most NK cells and also on tumor-infiltrating CD8 T cells, where it contributes to tumor-mediated inhibition (*9*, *10*). In contrast, KIR-only NK cells have the unique ability to sense down-regulation of single classical HLA class I genes by expression of a cognate inhibitory KIR, which closes a gap in the NK cell repertoire by complementing the HLA-E–specific NKG2A^+^ NK cells (*11*). Phenotypically, cILC1s lack expression of CD34 and CD117, which distinguishes them from established CD34^+^ NKPs identified in fetal liver (*12*), bone marrow (BM) (*13*), and CB (*14*), and CD34^−^CD117^+^ NKPs residing in secondary lymph nodes (SLNs) (*15*). These NKPs are primarily generating KIR^−^NKG2A^+^ and KIR^+^NKG2A^+^ NK cells, using various protocols with or without the use of feeder cells (*14*, *16*). Moreover, also ex vivo–isolated KIR-only NK cells tend to up-regulate NKG2A in culture, making the expansion of KIR-only NK cells in vitro difficult (*17*).

Notably, KIR-only NK cells hold substantial promise for adoptive NK cell therapy since they have the potential to eradicate allogeneic tumor cells based on their expression of licensed KIR that can be deliberately mismatched against a patient’s HLA class I type (*18*). The clinical relevance of this approach was previously shown in the setting of haploidentical stem cell transplantation, where KIR-only NK cells are suggested to play crucial roles in promoting the desired graft-versus-leukemia effect (*19*, *20*). A similar concept appears to be conceivable by using adaptive NK cells that share the KIR^+^NKG2A^−^ feature with KIR-only cells but additionally express the HLA-E–specific stimulatory receptor NKG2C^+^. However, adaptive NK cells can only be expanded from selected human cytomegalovirus (HCMV)^+^ donors that already have high frequencies of KIR^+^NKG2A^−^NKG2C^+^ NK cells (*21*). Together, an improved understanding of the in vivo developmental steps and the kind as well as location of NKPs leading to the generation of KIR-only NK cells appears to be highly desirable.

cILC1s with NKP function exhibit expression of T cell–associated molecules such as CD6, CD2, CD28, CD4, and CD8 and could be separated into two different subsets: a major CD5^+^ subset, which showed higher coexpression of T cell–associated molecules and failed to produce interferon-γ (IFN-γ) upon short-term cytokine stimulation and a minor CD161^+^ subset, which was able to secrete IFN-γ (*7*). On the basis of the close phenotypic and functional relationship between cILC1s, in particular the CD5^+^ subset, and T cells, we hypothesized that they might share a developmental link (*7*). This notion is compatible with the observation that CD5^+^CD161^−^ cILC1s significantly decrease from newborns (0 to 1 years), an age where thymic involution begins (*22*), to school children (6 to 12 years) and teenagers (13 to 18 years) (*23*).

The human thymus constitutes the organ where CD34^+^ lymphoid precursors coming from the BM are realizing a T cell fate. The journey starts within the thymic cortex, where BM-derived CD34^+^CD4^−^CD8^−^ lymphoid precursors go through consecutive double-negative (DN) stages. At the DN stages, CD34^+^ precursors still have the ability to commit to either NK cell (*24*–*26*), ILC2 (*27*), or γδ T cell (*28*) fates. Furthermore, in vitro studies suggested the presence of a bipotent NK/T precursor within early DN stages in fetal thymus in human as well as mouse (*29*), which are characterized by overexpression of ID3 (*25*) and in murine fetal liver (*30*). In this context, ID3 expression has been described to be a functional switch during thymic development of the DN stages as high ID3 expression favors NK development over T cell development (*25*). ID3 was shown by us to be highly expressed in cILC1s from neonates but not adults (and also not in tILCs) (*5*), which is compatible with a potential thymic origin of CB-derived ILCs. After the DN3 stage, CD34 expression is down-regulated and T cell precursors become fully committed to the T cell fate by rearranging their T cell receptor β (TCRβ) chain. The cells become immature single positive (ISP) cells, which in humans are CD4^+^CD8^−^ (*31*), whereas they are CD4^−^CD8^+^ in mice (*32*). Next, in a step commonly referred to as β-selection checkpoint, the TCRβ chain is productively rearranged and successfully expressed together with a surrogate pre-Tα chain and ISPs become double positive (DP) for CD4 and CD8 (*33*). Eventually, DPs with productively rearranged TCRα chains start migrating through the outer cortex encountering cortical thymic epithelial cells (TECs). Once DP cells have been positively selected, they undergo negative selection eliminating dominantly autoreactive T cells (*34*). The DP cells then become single-positive (SP) CD3^+^ T cells, expressing either CD4 or CD8 (*31*, *33*) and down-regulating CD1a expression (*35*). Besides the various developmental T cell stages, human as well as murine thymus also harbors innate lymphocytes including NK cells (*36*) and all major ILC subsets (*37*–*39*).

ILCs have been originally described to lack rearranged antigen receptors (*40*). However, we and others were able to detect residual rearranged TCR segments via transcriptome analyses in cILC1s and tonsillar ILC1s, suggesting that both circulating and tissue ILC1s might have a thymic origin (*7*, *41*–*43*). Notably, dynamic changes of thymic ILCs (thyILCs) have been observed during murine neonatal development (*44*), but more detailed information is only available for the ILC2 subset: ILC2 precursors are thought to split very early during the DN T cell developmental stage from the T cell lineage. While the mouse ILC2 precursor requires RORα expression (*37*), in humans, the ILC2 fate seems to be predominantly regulated by Notch signaling (*27*). Of note, a prior study described thyILCs to express CD5 (*38*). Apart from these observations, human thyILCs have not been deeply characterized so far. In particular, it is unclear whether human ILCs migrate into the thymus to become tissue resident, as suggested by the “ILC-poiesis” concept (*45*) or whether they, similar to T cells, require thymic signals for their development on site.

Here, to investigate the role of the thymus for ILC development, we conducted a thorough analysis of ILCs from postnatal human thymi. We identify an ILC1-like NKP in the thymus, which we termed thyILC1, which can efficiently differentiate into KIR^+^NKG2A^−^ KIR-only NK cells displaying a clonally diversified KIR repertoire and mirroring the respective phenotype of primary NK cell in PB. thyILC1s are more abundant than thyILC2 and thyILC3 and also outnumber thymic NK (thyNK) cells. The putative thymic origin of cILC1s is reinforced by analysis of patients with *FOXN1* mutations, characterized by a dysfunctional thymic architecture, which show a profound lack of cILC1s. The study suggests that the thymus provides a supportive environment for the development of ILC1-like NKPs, which can efficiently differentiate into high frequencies of KIR-only NK cells, complementing previously identified CD34^+^CD117^+^ NKPs residing in BM and SLNs that preferentially generate “NKG2A-only” NK cells.

## RESULTS

### thyILC1s are the predominant innate lymphocyte subset in human thymus

**Fig. 1.**
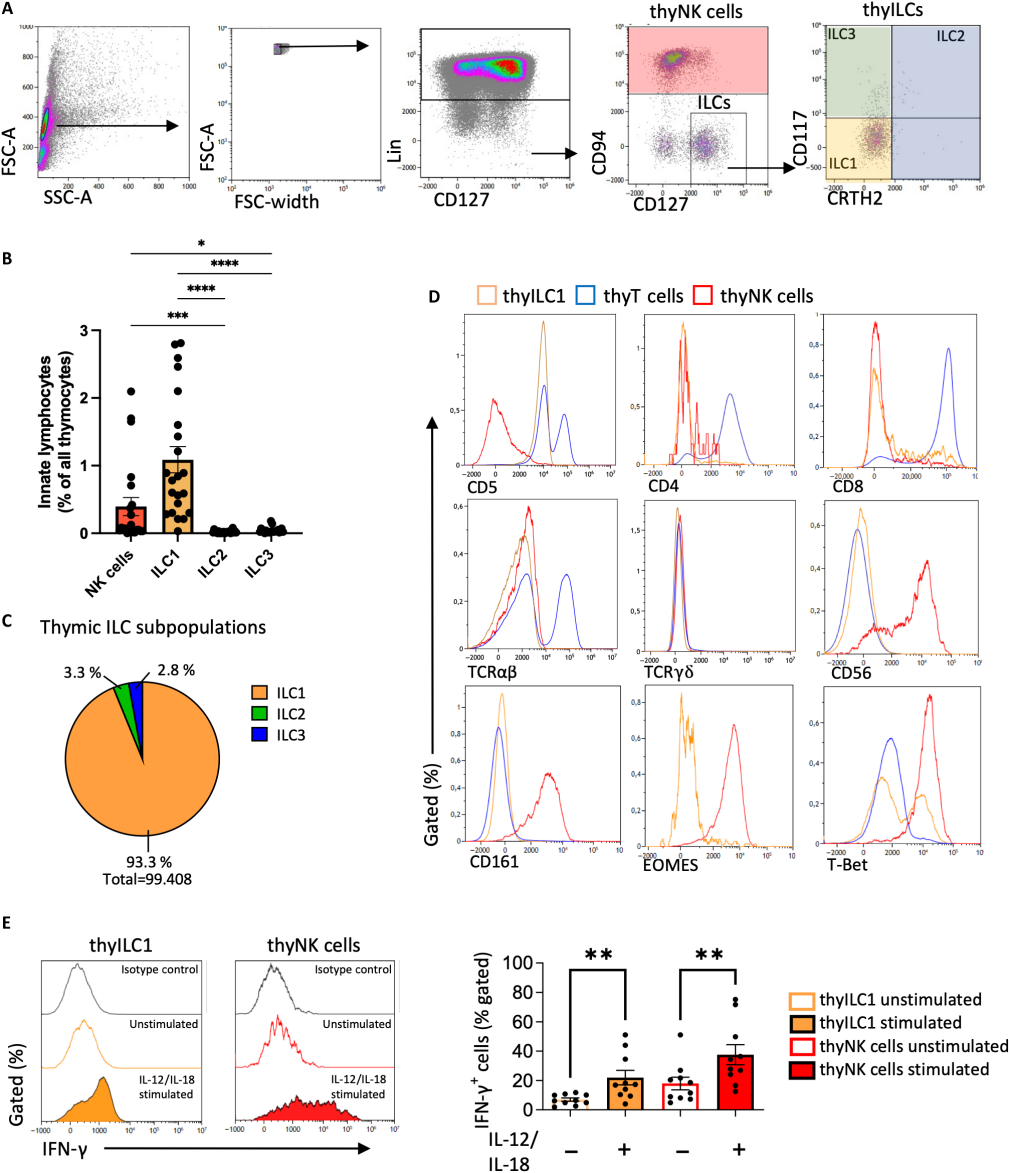
Human thyILC1s are the predominant innate lymphocytes subset and show a unique phenotype compared to thymic mature CD4^+^ T cells and NK cells. Thymocytes were isolated from fresh human postnatal thymi (PNT) (*47*) from pediatric patients in the need for cardiac surgery (*n* = 22; mean age, 6 months). Thymocytes were stained and analyzed via flow cytometry. (**A**) Exemplary gating strategy to identify NK cells (lin^−^CD94^+^), thyILC1s (CD117^−^CRTH2^−^), thyILC2s (CD117^+/−^CRTH2^+^), and thyILC3s (CD117^+^CRTH2^−^) in human PNT samples. (**B**) Bar graph showing the frequencies of NK cells (red), ILC1 (orange), ILC2 (green), and ILC3 (blue) from all thymocytes. (**C**) Pie chart displaying the mean frequency of thyILC1s (orange), thyILC2s (green), and thyILC3s (blue). (**D**) Representative histograms for T cell, NK cell, or ILC surface receptors for thyILC1s (yellow), thyNK cells (red), and thyCD4^+^ T cells (blue). (**E**) Representative histograms and bar graphs showing IFN-γ expression after 16-hour interleukine (IL) stimulation between thyILC1s (orange) and thyNK cells (red) compared to unstimulated controls and isotype controls (*n* = 10). Anti-CD11c antibody was added to the previously described lineage cocktail (*7*, *46*) for all plots, later also −CD4, −CD8 to ensure the exclusion of T cell stages, except for (D). The height of the bars represents the means ± SEM. Levels of significance were calculated with a Friedman test and Dunn’s multiple comparison (B) or with a Wilcoxon test between the stimulated and unstimulated condition (E), *P < 0.05, **P < 0.01, ***P < 0.001, and ****P < 0.0001. Forward scatter area (FSC-A); side scatter area (SSC-A).

The study was initiated to assess whether ILC1-like cells with NKP properties are present in the human thymus and to define their relationship to the previously identified cILC1s found in CB and PB (*7*). To this end, thymocytes were isolated from thymi of children undergoing cardiac surgery, which were otherwise immunologically healthy (*n* = 22; mean age, 6 months). To analyze thyILCs, we modified the lineage (lin) cocktail of our previously established flow cytometric staining panel for cILCs (*46*) by including CD11c to remove thymic dendritic cells (*47*). thyILCs were defined as Lin^−^(CD1a, CD3, CD4, CD8, CD11c, CD14, CD19, CD34, CD123, CD235a, FCER1a, TCRαβ, TCRγδ) CD94^−^CD127^+^ cells. The three ILC subpopulations were then defined as thyILC1s (CRTH2^−^CD117^−^), thyILC2s (CRTH2^+^CD117^+/−^), and thyILC3s (CRTH2^−^CD117^+^) (Fig. 1A). Notably, thyILC1s were much more frequent than thyILC2s and thyILC3s (Fig. 1B) and accounted for >90% of all thyILCs (Fig. 1C). They were also significantly more frequent than thyNK cells (Mann-Whitney test, thyILC1s versus thyNK cells, ***P = 0.0004), which represented the second most frequent population among innate thymocytes. Similar to the previously defined cILC1s in CB, thyILC1s homogenously expressed CD5 (at similar levels to thymic CD5^dim^ T cells) and lacked surface expression of molecules associated with mature T cells (TCRαβ, TCRγδ, CD4, and CD8), NK cells (CD56, EOMES, CD200R1, NKp80, NKp46, CD16, and KIR), and classical ILCs (CD161) (Fig. 1D and fig. S1). Furthermore, the ILC1-associated transcription factor (TF) T-bet was expressed in approximately 50% of thyILC1s (Fig. 1D).

A hallmark of ILC1s is the production of IFN-γ upon stimulation with proinflammatory cytokines (*48*). Thus, functional analyses were performed by short-term stimulation of thyILC1s with interleukin-12 (IL-12) and IL-18. Compared to unstimulated thyILC1s, a significant induction of IFN-γ production was observed, which was comparable to IFN-γ production seen in thyNK cells (Fig. 1E). Together, thyILC1s are the most abundant ILC type in the human thymus and exhibit ILC1-like phenotypic and functional properties.

### thyILC1s have NKP potential with a bias toward expression of KIRs

**Fig. 2.**
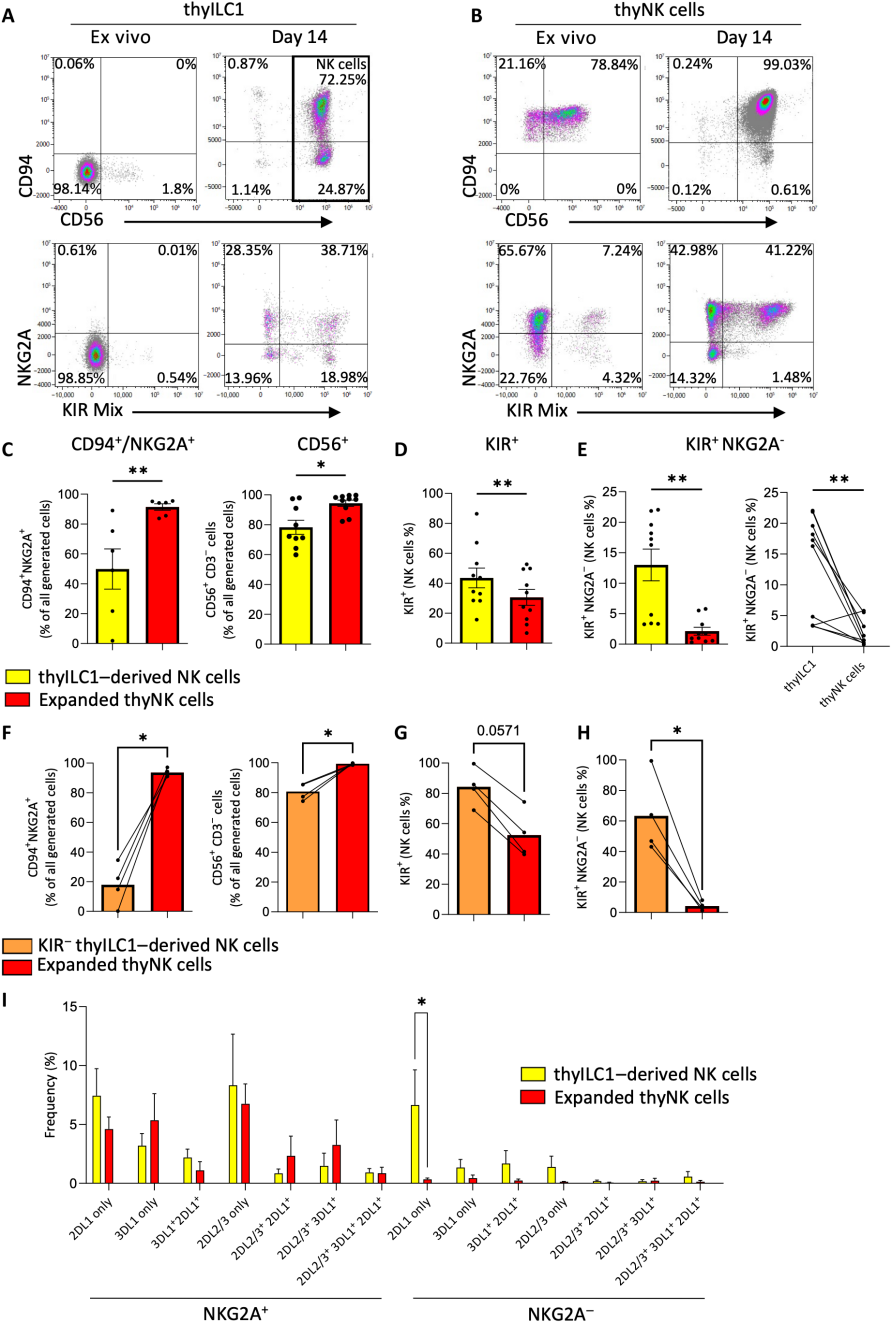
ThyILC1s show NKP potential to differentiate into KIR^+^ NKG2A^−^ NK cells. ThyILC1s and thyNK cells were flow cytometrically sorted and cultivated in vitro on OP9-DLL1 under NK cell differentiation supporting conditions (IL-2, IL-7, IL-15, SCF, and Flt3-L). After 14 days, the generated cells were analyzed for NK cell–specific surface receptors. Exemplary dot plots show the expression of CD94 against CD56 (top) and NKG2A against KIR-Mix (comprising antibodies for KIR2DL1/S1, KIR3DL1, and KIR2DL2/L3/S2) ex vivo (0 days) and after in vitro cultivation (14 days) of (**A**) thyILC1s and (**B**) thyNK cells. Bar graphs showing the frequencies of (**C**) CD94^+^NKG2A^+^ and CD56^+^ cells, (**D**) total KIR expression on total NK cells, and (**E**) KIR^+^NKG2A^−^ NK cells of the thyILC1-derived NK cells (yellow bar) and expanded thyNK cells (red bar) (*n* = 6 to 10). (**F** to **H**) KIR^+^ thyILC1s were specifically excluded by cell sorting. (F) Bar graphs showing the frequency of CD94/NKG2A^+^ and CD56^+^ cells in KIR^−^ thyILC1-derived NK cells (orange) and expanded thy NK cells (red) as well as the frequency of (G) total KIR^+^ and (H) KIR^+^NKG2A^−^ cells. (**I**) Dissection of the KIR repertoire of the thyILC1-derived NK cells (yellow bar) and expanded thyNK cells (red bar) analyzing the major inhibitory receptors KIR2DL1, KIR2DL2, KIR2DL3, KIR3DL1 ^+/−^NKG2A (*n* = 6). Data were generated from ≥3 independent experiments and ≥3 different donors (*n* = 6 to 12). The height of the bars represents the means ± SEM. Levels of significance were calculated with a Wilcoxon test, *P < 0.05, **P < 0.01.

Next, we assessed whether thyILC1s have the potential to differentiate into NK cells. For this purpose, we sorted thyILC1s and cocultivated them on OP9-DL1, a murine stroma cell line that expresses the Notch ligand DLL1 and was previously shown to support NK cell development from cILCs (*7*). Coculture with OP9-DL1 and the appropriate cytokines led to efficient differentiation of thyILC1s into NK cells (CD3^−^CD56^+^CD94^+^) (Fig. 2A). Control thyNK cells, expanded under the same conditions, kept their basic CD56^+^CD94^+^NK cell phenotype as expected (Fig. 2B). The frequency of NKG2A expression on thyILC1-derived NK cells was consistently lower compared to expanded thyNK cells (Fig. 2, A to C). Instead, receptors encoded by the KIR family were significantly more frequently expressed on thyILC1-derived NK cells than on expanded thyNK cells (43.55 versus 30.59%, **P = 0.0059) (Fig. 2D). Strikingly, the newly generated thyILC1-derived NK cells exhibited an unusually high frequency of KIR^+^NKG2A^−^ cells, whereas this subset was barely detectable in the cultures starting with thyNK cells from the same donors (13.00 versus 2.13%, **P = 0.0059) (Fig. 2E). Of note, a small fraction (~0.5%) of thyILC1s expressed KIR (Fig. 2A). Further analysis revealed that they represent a small subset of CD94^−^ NK cells that besides KIR also expressed Eomesodermin (EOMES) and NK cell markers such as CD56, CD16, NKp46, and NKp80 (fig. S2, A and B). To exclude these NK cells from the thyILC1 subset, in a subsequent experiment, KIR^+^ thyILC1s were gated out before cell sorting (fig. S2C). In the following differentiation experiment, these KIR^−^ thyILC1s showed a strong bias toward KIR^+^NKG2A^−^ NK cells (63.34% ± 12.35 versus 4.19% ± 1.46 starting from thyNK cells), proving that in the previous experiments, the contaminating CD94^−^KIR^+^ NK cells did not majorly contribute to the generation of KIR^+^NKG2A^−^ NK cells. On the contrary, they might have had a regulating influence since the frequency of KIR^+^NKG2A^−^ NK cells was higher without than with contaminating CD94^−^KIR^+^ NK cells (Fig. 2, F to H).

Analysis of the distribution of the inhibitory KIR receptors for the three major HLA class I ligands, C1 (KIR2DL2/3), C2 (KIR2DL1), and Bw4 (KIR3DL1) revealed that thyILC1-derived NK cells had developed a broad KIR repertoire, representing all possible KIR combinations. As illustrated in Fig. 2I, only thyILC1s, but not thyNK cells developed a diverse KIR repertoire in the absence of NKG2A expression.

Further phenotypic characterization showed that KIR^+^NKG2A^−^ thyILC1-derived NK cells uniformly expressed CD56, with some expressing CD94^+^, whereas no expression of NKG2C was observed (fig. S3A). Within the KIR^−^NKG2A^−^ fraction of the thyILC1-derived NK cells, all cells expressed CD117 with nearly half coexpressing NKp44 (fig. S3B).

**Fig. 3.**
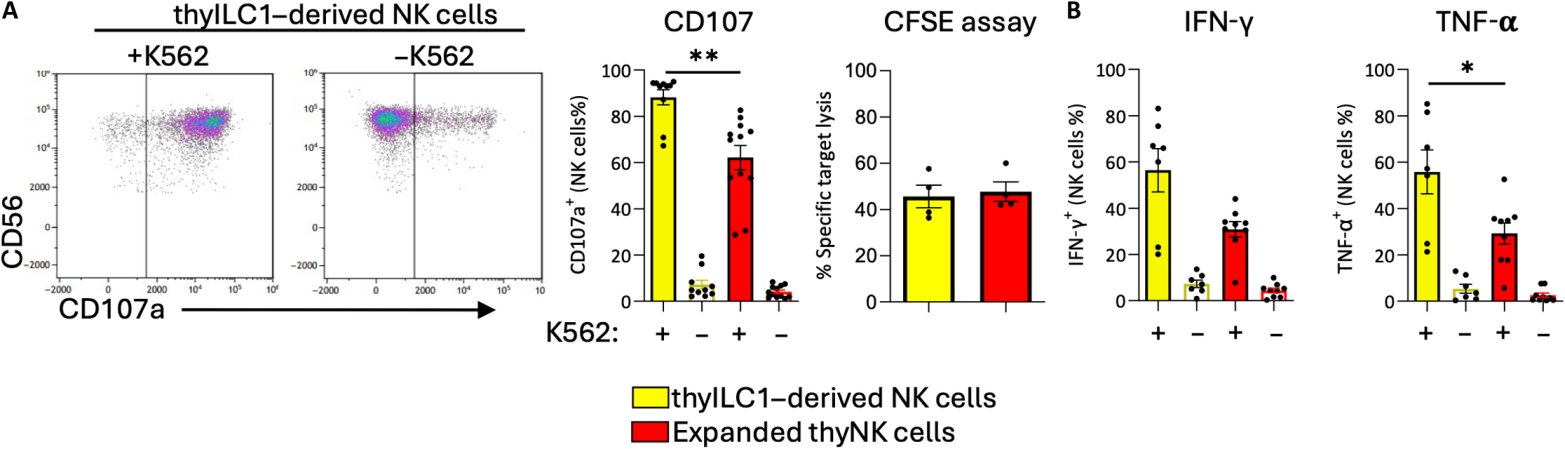
ThyILC1s differentiate into functional NK cells. Cells were cultivated as described in Fig. 2 and effector functions analyzed against the HLA class I–deficient cell line K562. (**A**) Exemplary dot plots showing the granule mobilization marker CD107a expression of thyILC1-derived NK cells after 5 hours of exposure to the HLA-class I–deficient cell line K562 in a 1:1 effector target ratio. Bar graphs showing CD107a expression with (+) or without (−) K562 for thyILC1-derived NK cells (yellow bar, *n* = 10) compared to expanded thyNK cells (red bar, *n* = 12) and specific target cell lysis measured by a CFSE/PI-based cytotoxicity assay, shown in a bar graph for thyILC1-derived NK cells (yellow) and expanded thyNK cells (red) (*n* = 4). (**B**) Bar graphs showing the intracellular production of IFN-γ and TNF-α for thyILC1-derived NK cells (yellow bar, *n* = 7) compared to expanded thyNK cells (red bar, *n* = 10). Data were generated from at least three independent experiments with at least three different donors (*n* = 6 to 12). The height of the bars represents the means ± SEM. Levels of significance were calculated with a Wilcoxon test, *P < 0.05, **P < 0.01.

Last, we determined the functional properties of thyILC1-derived NK cells. Strong degranulation, measured as mobilization of surface CD107a to the cell surface, was noted upon stimulation of thyILC1-derived NK cells with the HLA class I–deficient target cell line K562. The response was significantly higher than the increase seen in CD107a on expanded thyNK cells (88.23 versus 62.16%, ****P < 0.0001) (Fig. 3A). However, the target-specific lysis, as determined by the carboxyfluorescein diacetate succinimidyl ester/propidium iodide (CFSE/PI)–based killing assay, was comparable between the two groups (45.59 ± 4.92% for thyILC1-derived NK cells versus 47.68 ± 4.24% for expanded thyNK cells) (Fig. 3A). Furthermore, a strong cytokine response of IFN-γ and TNF-α was elicited by thyILC1-derived NK cells, which was higher compared to expanded thyNK cells (Fig. 3B). Together, the data demonstrate that ILC1s with NKP potential are present in the thymus. thyILC1s have the ability to efficiently differentiate into mature NK cells that have down-regulated NKG2A and are characterized by a broad KIR repertoire as well as high effector functionality.

### The transcriptional signature of thyILC1s is distinct from other thymocyte subsets

**Fig. 4.**
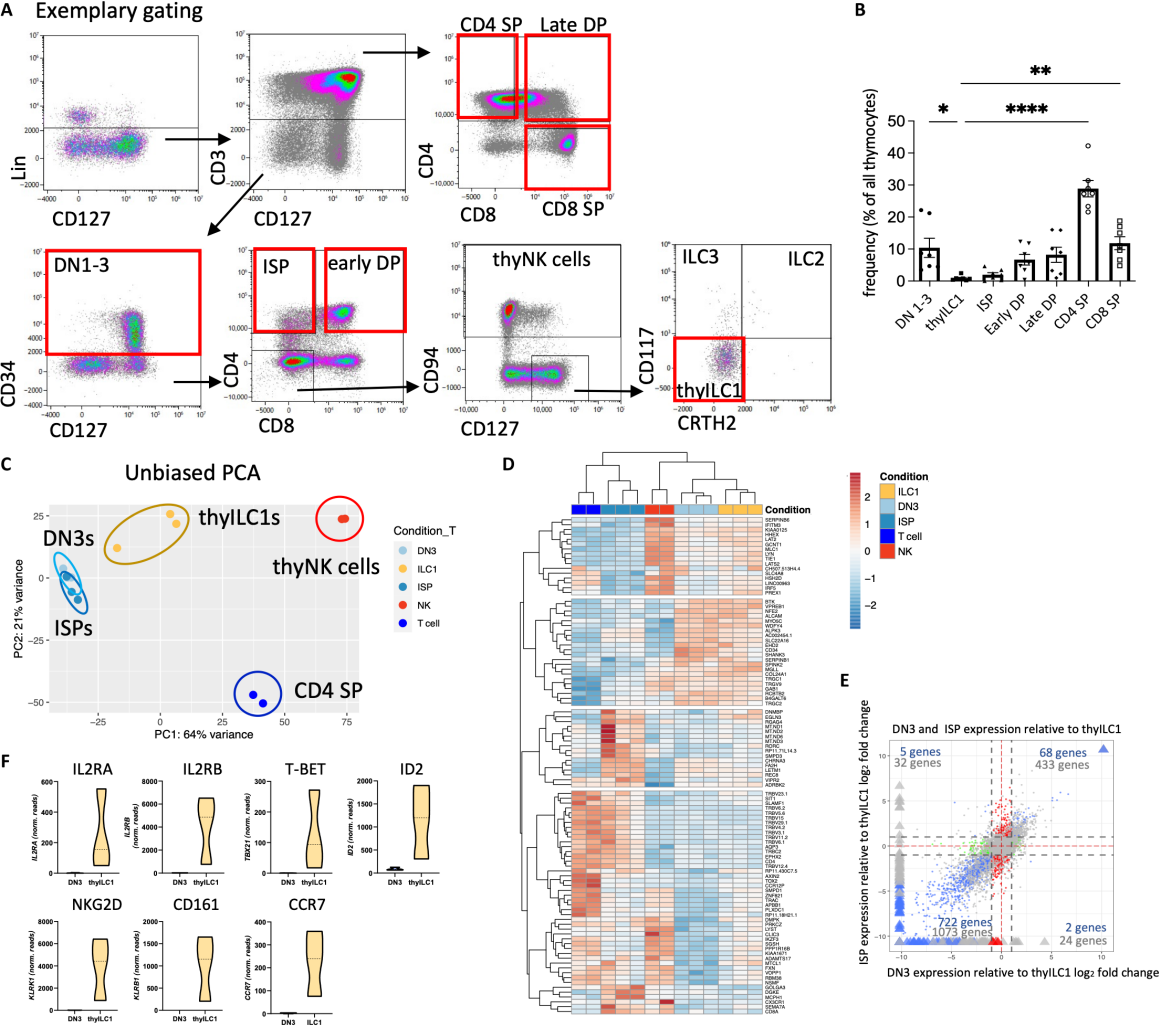
ThyILC1s are a phenotypically and transcriptionally distinct subset within the human thymus. (**A**) Exemplary gating strategy to identify the frequencies of thyILC1s with different thymic T cell progenitor and committed stages. Lin^−^ cells were separated according to their CD3 surface expression. Lin^−^ CD3^+^ T cells were categorized into three distinct groups: late DPs (CD4^+^CD8^+^), single-positive CD4^+^ (CD4 SP), and single-positive CD8^+^ (CD8 SP) T cells. In contrast, Lin^−^CD3^−^ cells were further divided into CD34^+^ double-negative (DN1-3) cells. Within the Lin^−^CD3^−^CD34^−^ thymocyte population, two subsets were identified: ISPs (CD4^+^CD8^−^) and early DPs (CD4^+^CD8^+^). To ultimately identify thyILC1s, Lin^−^CD3^−^CD34^−^CD4^−^CD8^−^ cells were further defined as CD94^−^CD127^+^CD117^−^CRTH2^−^. (**B**) Frequencies of the defined thymocyte subsets in a bar chart (*n* = 6). (**C**) Unbiased PCA of bulk RNA-seq data from flow cytometrically sorted ISPs (blue), DN3s (light blue), thyILC1s (yellow), thyNK cells (red), and thy CD4^+^ SP T cells (dark blue). (**D**) Heatmap of the top 100 differentially expressed genes between ISPs (blue) and DN3s (light blue) including CD4^+^ SPs T cells (dark blue), thyNK cells (red), and thyILC1s (yellow) (*n* = 2 to 3). (**E**) Four-way plot of bulk RNA-seq data from thyILC1s, DNs and ISPs (*n* = 3). (**F**) Violin plots of the normalized read counts of selected NK cell and ILC related genes from DN3 and thyILC1 (*n* = 3). The height of the bars represents the means ± SEM. The levels of significance were calculated with a nonparametric analysis of variance (ANOVA) (Kruskal-Wallis Test) with multiple comparison posttest between all populations (B), *P < 0.05, **P < 0.01, and ****P < 0.0001.

While thyILC1s could be readily distinguished from other ILC subsets in the thymus, it was so far unclear how they are phenotypically and transcriptionally related to the different thymic T cell developmental stages. To this end, we modified our flow cytometric strategy to include T cell markers previously being part of the Lineage cocktail (Fig. 1A). As shown in Fig. 4A, on the basis of surface expression of CD127 and lack of CD3, CD4, CD8, CD94, and CD34, thyILC1s could be clearly separated from DN1-3s (Lin^−^CD3^−^CD34^+^), ISPs (Lin^−^CD3^−^CD34^−^CD4^+^), early DPs (Lin^−^CD3^−^CD34^−^CD4^+^CD8^+^), and the more mature CD3^+^ T cell stages (late DP, CD4SP, and CD8SP T cells). In terms of frequencies, thyILC1s were the least abundant population among these subsets in the descending order: SPs > DPs > DNs > ISPs > thyILC1s (Fig. 4B).

Next, we performed global transcriptome analyses of flow cytometrically sorted thyILC1s, DN3s, ISP, CD4^+^ SP, and thyNK cells (fig. S4, A to C for gating). Unbiased principal components analyses (PCA) showed distinct clustering of thyILC1s apart from CD4^+^ SP T cells as well as thyNK cells with closest similarity to DN3s and ISPs (Fig. 4C). To more clearly define the transcriptional relationships of thyILC1s to the latter two subsets, we assessed at the most differentially expressed genes between DNs and ISPs with inclusion of thyILC1s, CD4 SP T cells, and thyNK cells after hierarchical clustering in a heatmap. As shown in Fig. 4D, thyILC1s were most closely related to CD34^+^CD1a^+^ DN3s and clearly separated from ISPs. Whereas, as expected, ISPs showed closest resemblance to CD4 SP T cells, thyNK cells clustered separately. Furthermore, direct comparison of thyILC1s with DN3s and ISPs in a log_2_ fold change plot revealed a comparatively high number of genes (722 genes) that are significantly overexpressed in thyILC1s compared to DN3s and ISPs (Fig. 4E). A preponderance of overexpressed genes compared to DN3 is also illustrated in a respective heatmap of thyILCs versus DN3s (fig. S5). Notably, thyILC1s showed higher expression of ILC1- and NK cell–related genes such as *TBX21* (TBET), *KLRB1* (CD161), *ID2*, the chemokine receptor *CCR7*, *KLRK1* (NKG2D), and the IL-2 receptor–specific chains IL2RA and IL2RB than DN3s (Fig. 4F). Of note, we did not observe an *ID3/ID2* ratio > 1 (fig. S5B) as previously seen within cILC1 (*7*). Among the few genes significantly overexpressed in DN3s compared to thyILC1s were *CD1a*, *CD1b*, and *CD1c* (fig. S5A), characteristic for cortical thymocytes (*49*) and in case of CD1a tightly associated with commitment of CD34^+^ DNs toward the T cell lineage (*31*, *35*).

### thyILC1 express the preTCRα chain in the absence of rearranged TCRβ chains

**Fig. 5.**
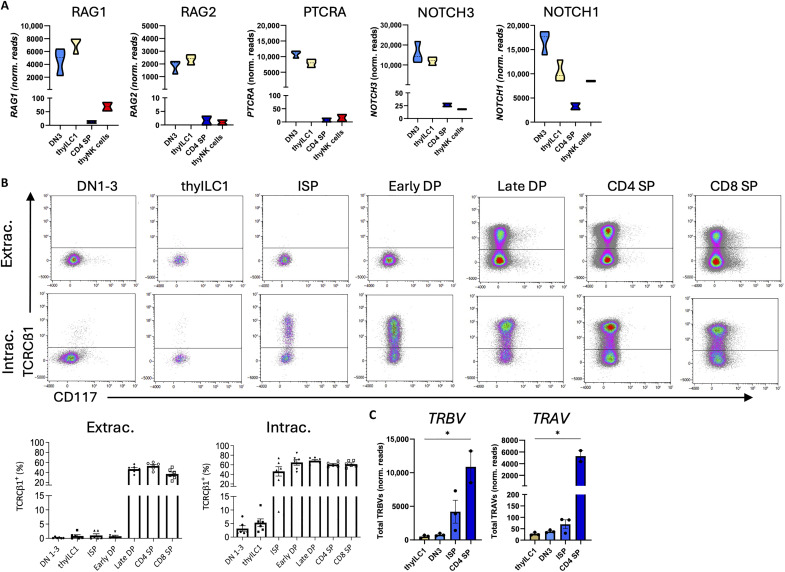
thyILC1 express the preTCRα chain without rearranged TCRβ chains. For the gating, please read the legend of Fig. 4. (**A**) Violin plots of the normalized read counts of selected T cell development–related genes for DN3, thyILC1, thyNK cells, and CD4^+^ SP T cells (*n* = 2 to 3). (**B**) Representative dot plots and quantification in a bar chart for extracellular (top) and intracellular (bottom) expression of the TCR constant β1 (TCRCβ1) chain from the (Fig. 4A) defined populations (T cell stages and thyILC1s, *n* = 6). (**C**) Bar charts of the normalized read counts of total T cell receptor beta variable (*TRBV*) and T cell receptor alpha variable (*TRAV*) chain genes from bulk DESeq2 normalized RNA-seq data of DN3, thyILC1s, ISPs, and CD4^+^ SP T cells (*n* = 2 to 3). The height of the bars represents the means ± SEM. The levels of significance were calculated with a nonparametric ANOVA (Kruskal-Wallis Test) with multiple comparison posttest with thyILC1s compared all other cells (C), *P < 0.05.

Notably, similar to DN3s, thyILC1s expressed *RAG1* and *RAG2*, constituting key components of TCR somatic rearrangement as well as *PTCRA*, encoding the preTCRα chain (Fig. 5A). Assembly of a productive preTCR complex (consisting of preTCRα, TCRβ, and CD3 components) together with Notch signaling are key requirements for passing the β-selection checkpoint that controls selection of suitable recombined TCRβ chains (*31*). Since thyILC1s strongly expressed *NOTCH1* and *NOTCH3* (Fig. 5A), the question arose whether thyILC1s would already express the preTCR complex.

Since no suitable reagents are available for specific detection of the human preTCRα chain via flow cytometry, we used a TCR constant β1 (TCRCβ1) chain–specific antibody, which stains all TCRβ chains that have incorporated the TCRCβ1 segment instead of the TCRCβ2 segment (*50*). As outlined in Fig. 5B, in thyILC1s as well as in DN3s and ISPs, no expression of TCRCβ1 could be observed on the cell surface thereby excluding the presence of surface preTCR complexes. In general, extracellular TCRCβ1 expression correlated with high CD3 expression on the cell surface, which was first detectable at the late DP stage. In contrast, intracellular TCRCβ1 was already present in ISPs but rare in DN3s and thyILC1s (Fig. 5B). Similar to DNs, thyILC1s showed intracellular expression of CD3ε and CD3δ chains in ~40 and ~90% of cells, respectively, but no expression on the cell surface (fig. S6). Consistent with the analysis of TCRCβ1 proteins, expression of rearranged TCR Vβ chains (Fig. 5C) was very low in DN and thyILC1 subsets, whereas ISPs showed intermediate and CD4 SP T cells strong transcriptional activity (fig. S7A for individual TRBV genes). In line with their early T cell developmental stage, expression of rearranged TCR Vα segments (Fig. 5C) was very low to undetectable in thyILC1s and DNs and still rare in ISPs compared to strong expression in CD4 SP T cells (fig. S7B for individual TRAV genes). Together, thyILC1s were shown to have activated a transcriptional program including *RAG1*, *RAG2*, *PTCRA*, and *NOTCH*, that principally enables initiation of TCR rearrangement, but failed to do so, precluding assembly of preTCR complexes.

### thyILC1s are unable to differentiate into T cells

**Fig. 6.**
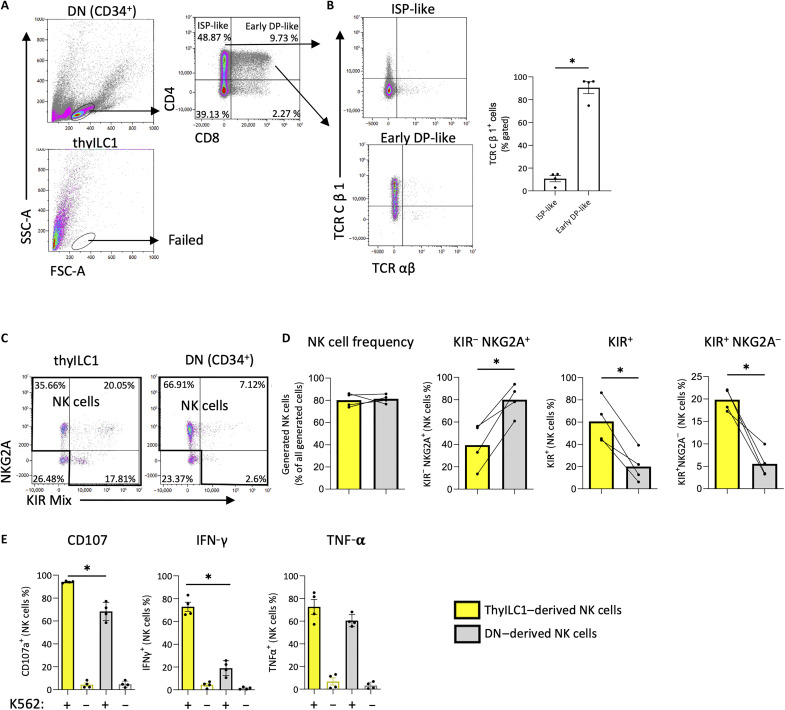
ThyILC1s show NK cell but no T cell differentiation potential. DN thymocytes (CD34^+^) and thyILC1s were flow cytometrically sorted and cultivated in parallel in vitro on OP9-DLL1 under either T cell [IL-7 and Flt3-L, (**A** and **B**)] or NK cell differentiation supporting cytokines (Fig. 2), (**C** to **E**) for 14 days (*n* = 3 to 4). The cells were flow cytometrically analyzed. (A) Representative dot plots showing FSC-A and SSC-A for thyDN-derived cells (top) and thyILC1-derived cells (bottom). ThyDN-derived cells were further gated for CD4 and CD8 expression. (B) Representative dot plots for TCRαβ and TCRCβ1 expression of two thyDN-derived populations: CD4^+^CD8^−^ ISP–like and CD4^+^CD8^+^ DP–like are shown. Bar graphs showing the expression TCRCβ1 within CD4^+^CD8^−^ ISP–like and CD4^+^CD8^+^ DP–like cells (*n* = 4). (C) Representative dot plots showing NKG2A against KIR-Mix (comprising antibodies for KIR2DL1/S1, KIR3DL1, and KIR2DL2/L3/S2) for thyILC1- and DN-derived cells (3000 events displayed). NK cells were defined as expressing either NKG2A or KIR or both. (D) Bar graphs showing the frequency of generated NK cells and NK cell subsets (KIR^−^NKG2A^+^, KIR^+^NKG2A^+^, and KIR^+^NKG2A^−^) from thyILC1s (yellow bar) or DNs (gray bar). (E) The functionality of the generated cells was analyzed via the HLA class I–deficient cell line K562. Bar charts of surface CD107a expression, intracellular IFN-γ and TNF-α with (+) or without (−) K562 for thyILC1-derived NK cells (yellow bars) in comparison to DN-derived NK cells (gray bars). The height of the bars represents the mean. Each dot represents a different donor. The lines represent the levels of an individual donor for each NK cell population (C). Levels of significance were calculated with a Wilcoxon test; for (E), statistics were calculated between the two groups with added K562 (+), *P < 0.05.

Since thyILC1s expressed genes associated with V(D)J rearrangement (Fig. 5A), we investigated whether they might constitute bipotent progenitors with the ability to differentiate into NK as well as T cells. To this end, thyILC1s as well as DNs were flow cytometrically sorted and cocultured in parallel on OP9-DL1 using cytokines (IL-7 and Flt3-L) that support T cell development (*51*). As expected, under these conditions, DNs proliferated and progressed to the more mature CD4^+^ ISP (~50%) and CD4^+^CD8^+^ early DP (~10%) stages (Fig. 6A). The in vitro–generated early DPs showed significant higher expression of TCRCβ1 compared to the ISPs, consistent with further progression of early DPs across the β-selection checkpoint (Fig. 6B). In contrast, thyILC1s did not proliferate or differentiate under these conditions and, at the end of the 2-week culture period, no cells with lymphocyte morphology were detectable anymore (Fig. 6A). Thus, our data suggest that thyILC1s have lost their T cell differentiation potential, at least in these experimental conditions.

### DNs and thyILC1s are two kinds of thymic NKPs with diverging differentiation capacity

Human DN thymocytes were previously reported to have NK cell differentiation potential (*29*), but the kinds and frequencies of KIR and NKG2 receptors induced on DN-derived NK cells are unknown. Thus, we next aimed to determine whether the ability to efficiently differentiate into KIR^+^NKG2A^−^ NK cells is unique to thyILC1s (Fig. 2) or whether NK cells generated from DNs show a similar bias toward expression of KIRs. To this end, thyILC1s and DNs were isolated from the same donors and cultured in parallel in NK cell differentiation conditions. DN thymocytes could be differentiated to NK cells with similar efficiency compared to thyILC1s: In both cases, about 80% of all generated cells were phenotypically identified as NK cells after 14 days of culture (Fig. 6, C and D). However, NK cells generated from DN thymocytes exhibited significantly lower frequencies of KIR^+^NK cells and in turn significantly higher frequencies of NKG2A^+^NK cells compared to thyILC1s (Fig. 6D). Furthermore, the KIR^+^NKG2A^−^ subset differentiated from DN thymocytes was significantly smaller than that from thyILC1-derived NK cells. In addition to this, thyILC1-derived NK cells exhibited a significantly higher functionality, assessed by cytotoxic granule mobilization (CD107a) and cytokine production (IFN-γ, not significant for TNF-α) than NK cells derived from DN cells (Fig. 6E). This is reminiscent of the stronger effector functions of thyILC1-derived NK cells in comparison to resident thyNK cells (Fig. 2). Together, the data suggest that the thymus might harbor two kinds of NKPs that generate complementary NK cell subsets: On the one hand CD34^+^ DN thymocytes are biased to develop into NKG2A-only NK cells and on the other hand thyILC1s being more biased toward development of KIR-only NK cells, characterized by a broad KIR repertoire.

### thyILC1 are transcriptionally distinct from thyNK cells in scRNA-seq analysis

**Fig. 7.**
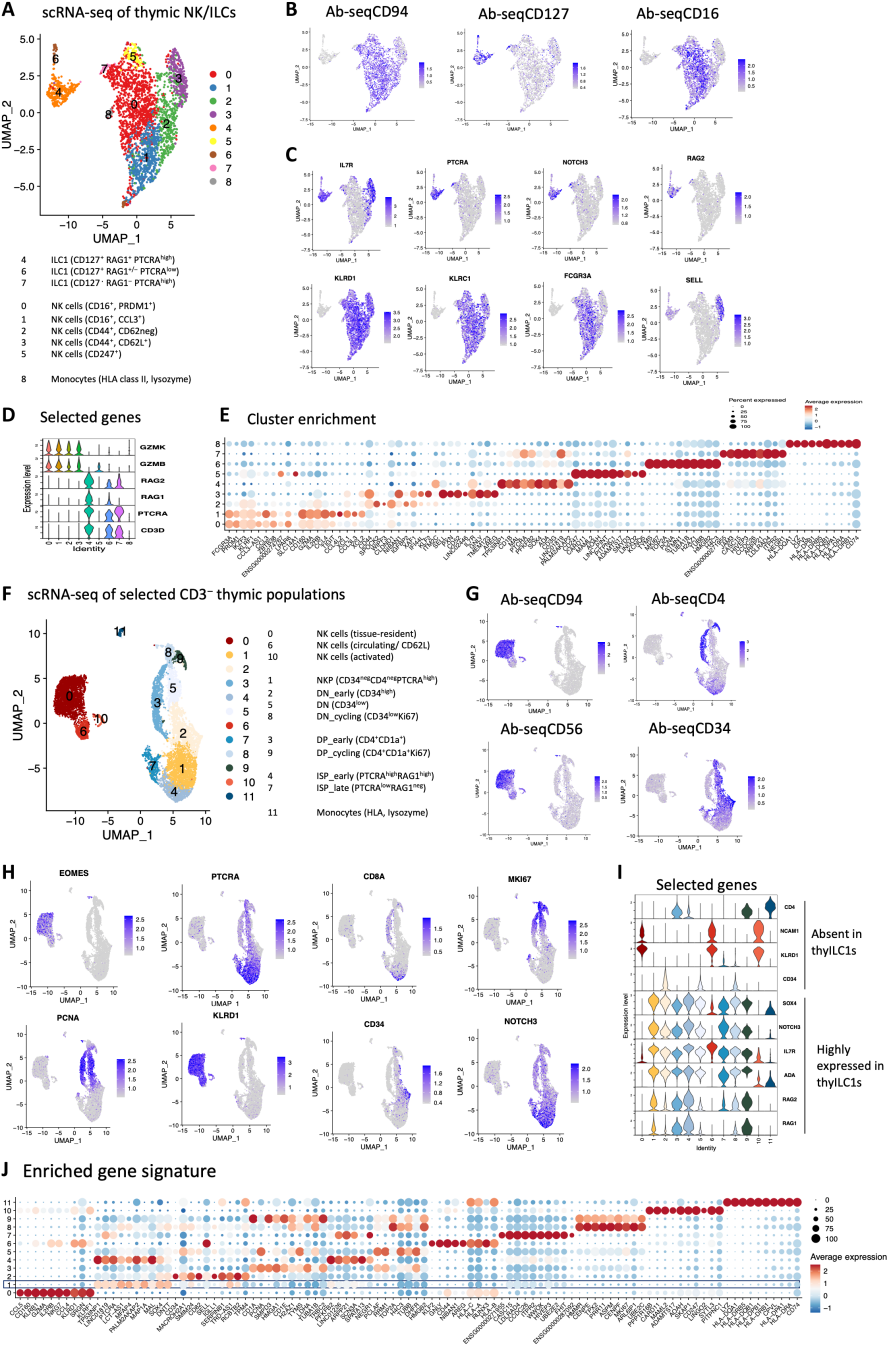
Single-cell transcriptional analysis distinguishes thyILC1s from NK cells and reveals similarities to DN thymocytes. Thymocytes were isolated from thymic samples (*n* = 3) and flow cytometrically sorted on three populations: 1) ILCs and NK cells (Lin^−^CD3^−^CD1a^−^CD34^−^CD4^−^CD8^−^CD127^+/-^CD94^+/−^ cells), 2) early CD3^−^ committed T cell stages (Lin^−^CD3^−^CD34^−^CD4^−/+^CD8^+/−^), and 3) DNs (Lin^−^CD3^−^CD34^+^) with subsequent AbSeq staining and scRNA-seq analysis. (**A**) UMAP visualization of the 1) population comprising thyILC and NK cells selectively with color coding of clusters 0 to 8. Feature plots showing (**B**) CD94, CD127, and CD16 surface expression determined by AbSeq antibodies and (**C**) the gene expression of selected genes: *IL7R*, *PTCRA*, *NOTCH3*, *RAG2, KLRD1*, *KLRC1*, *FCGR3A*, and *SELL.* (**D**) Violin plots showing *GZMK*, *GZMB*, *RAG2*, *RAG1*, *PTCRA*, and *CD3D* expression for each cluster. (**E**) Dot plot showing the top 10 enriched genes within each cluster. (**F**) UMAP visualization of all three sorted CD3^−^ thymic populations color coded by clusters 0 to 11. (**G**) Feature plots showing CD94, CD4, and CD56 surface expression determined by AbSeq antibodies and (**H**) expression of selected genes (*EOMES*, *PTCRA*, *CD8A*, *MKI67*, *CD34*, *NOTCH3*, *PCNA*, and *KLRD1*) to identify the different thymocyte populations. (**I**) Violin plots showing *CD4*, *NCAM1*, *KLRD1*, *CD34*, *SOX4*, *NOTCH3*, *IL7R*, *ADA*, *RAG2*, and *RAG1* expression for each cluster. (**J**) Dot plot showing the top 10 enriched genes for each identified cluster.

Given that the newly defined thyILC1 subset exhibited NKP potential, we next aimed to find out whether the transcriptomes of thyILC1s and thyNK cells are clearly distinguishable from each other on the single-cell level or whether they are rather in a transcriptional continuum. We thus conducted whole-transcriptome single-cell RNA sequencing (scRNA-seq) analysis of CD3-depleted thymocytes (three infants with ages ≤3 weeks), which were further enriched for thyILC1s and thyNK cells by flow cytometric cell sorting (lin^−^CD3^−^CD1a^−^CD34^−^CD4^−^CD8^−^CD127^+^ cells; fig. S4D). The pooled samples were tagged with an AbSeq panel consisting of 36 antibodies specific for various lineage antigens (table S1) plus additional AbSeq antibodies (described in section: scRNA-seq using BD rhapsody). thyILC1s and thyNK cells were located apart from each other by Uniform Manifold Approximation and Projection (UMAP) (calculated on the basis of whole-transcriptome analyses, Fig. 7A) and AbSeq (Fig. 7B) analyses: Three clusters corresponded to CD127^+^CD94^−^ thyILC1s (clusters 4, 6, and 7) and five clusters to CD127^−^CD94^+^ thyNK cells (clusters 0, 1, 2, 3, and 5). The thyILC1 clusters are characterized by expression of *IL7R* (CD127), *PTCRA*, *NOTCH3*, and *RAG2* (Fig. 7C), consistent with the data obtained by bulk RNA-seq analysis (Figs. 4 and 5). Among the genes that are expressed in NK cells but not thyILC1s are *GZMB*, indicative of mature NK cell subsets (clusters 0 to 3) and *GZMK*, typically expressed in immature NK cells (cluster 5) (Fig. 7D). thyILC1s are further subdivided into a major subcluster of supposedly resting thyILC1s (subcluster 4), and a small subset (subcluster 6) of proliferating thyILC1s, as indicated by high expression of *MKI67* (Ki67) and *PCNA* (Fig. 7E). The major thyILC1 cluster 4 showed further gene enrichment of *ADA* and the TF *SOX4* (Fig. 7E). Last, subcluster 7 constituted a rare CD127^+^ subset, which was characterized by high expression of *PTCRA* but was distinguishable from conventional thyILC1s by weak expression of *RAG1* (Fig. 7, A and E).

All thyNK clusters expressed *KLRD1* (CD94) as well as its respective binding partner encoding *KLRC1* (NKG2A), together identifying them as NK cells (Fig. 7B). Among thyNK cells, clusters 0 and 1 were both characterized by expression of *FCGR3A* (CD16), consistent with surface expression of CD16 detected by AbSeq staining (Fig. 7, B and C) and the TF *PRDM1* (a homolog of Hobit) and were distinguished from each other by expression of *CD160* and the chemokine receptors *CCR3* and *CCR4* in cluster 1 (Fig. 7D). Clusters 2 and 3 were both expressing *CD44* and were distinguished by expression of *SELL* (CD62L) in cluster 2, but not 3. Cluster 5 was characterized by strong expression of *CD247*, encoding the CD3 ζ chain, which is associated with the TCR complex in T cells but also serves signal transduction functions in NK cells (Fig. 7D). Last, cells in cluster 8 exhibited characteristics of monocytes due to high expression of HLA class II genes and *LYZ* (lysozyme) and the absence of NK cell (e.g., granzymes) as well as thymocyte markers such as CD3 (Fig. 7, D and E).

Together, thyILCs constitute a comparatively homogenous population of CD127^+^RAG1^+^PTCRA^+^ cells that can be further distinguished in a large resting and a small proliferating subset. thyNK cells are transcriptionally distinct and can be divided into five different subpopulations. The functional significance of this distinction into five thyNK cell subsets and the role of expression differences in PRDM1, CD44, CD160, and CD247 between them needs to be addressed in future studies.

### thyILC1 are embedded in the thymic transcriptional landscape between DN and ISP stages

Having established that thyILCs are readily distinguished from thyNK cells on the transcriptional level, we next wanted to locate thyILC1s within the broader transcriptional landscape of early thymic CD3^−^ T cell developmental stages. For this purpose, scRNA-seq analyses were extended by inclusion of all CD3^−^ early thymocytes including DNs, ISPs, and early DPs (fig. S4D) with a total of 8842 cells for downstream transcriptional analyses. A total of 12 subpopulations (clusters 0 to 11) could be distinguished by clustering and subsequently mapped onto the UMAP. Cells were organized in three clearly separated cell entities: a large thymocyte entity, composed of thyILC1s and various stages of DNs, ISPs, and DPs (clusters 1 to 5 and 7 to 9); an NK cell entity (clusters 0, 6, and 10); and monocytes (cluster 11) (Fig. 7F).

ThyILC1s were located in cluster 1, characterized by high levels of *PTCRA*, *NOTCH3*, and *RAG1/2*; absence of *CD34*; and lack of surface expression of CD94, CD56, and CD4 (Fig. 7, G to I). Notably, similar to the previous analysis (Fig. 7E), thyILC1s expressed *SOX4* at higher levels than all other thymic subsets, pointing to a possible role of SOX4 for lineage specification of the thyILC1 subset (Fig. 7, I and J). thyILCs were located adjacent to cluster 2, which exhibited high expression of CD34, compatible with early DNs, and constituting one of three DN subclusters (2, 5, and 8). The other two DN clusters showed high *PCNA* and low CD34 expression (DN CD34^low^), while cluster 8 likely constitutes cycling DNs, exhibiting high levels of *MKI67* (Fig. 7, I and J).

Furthermore, the other cluster neighboring thyILC1s were identified as ISPs (cluster 4). ISPs were majorly found in clusters 4 and 7, which both expressed CD4 on the cell surface by AbSeq analyses and on the basis of *PTCRA* and *RAG1* expression, could be further specified as early ISPs (subcluster 4, PTCRA^high^RAG1^high^) and late ISPs (subcluster 7, PTCRA^low^RAG1^neg^) (Fig. 7, G to J). DPs were located in clusters 3 and 9 on the basis of AbSeq staining for CD4, transcription of *CD8A* and *CD1a*, and reduced *PTCRA* expression (Fig. 7, G and H). DPs were further specified as early DPs (CD4^+^CD1a^low^) and late DPs (CD4^+^CD1a^+^), respectively, with the latter exhibiting high expression of the proliferation marker Ki67 (*MKI67*) (Fig. 7, H and J).

Last, NK cells were identified in clusters 0, 6, and 10, which were clearly separated from the rest of the cells and were characterized by AbSeq expression of CD56 and transcription of *EOMES*, *KLRD1* (CD94), *KLRB1* (CD161), *KLRF1* (NKp80), and the TF *RUNX3* (Fig. 7, G to J). Together, scRNA-seq analyses revealed thyILC1s to be embedded within early T cell developmental stages, with closest similarities to DNs and ISPs.

### cILC1 are significantly reduced in patients with inborn thymic defects

**Fig. 8.**
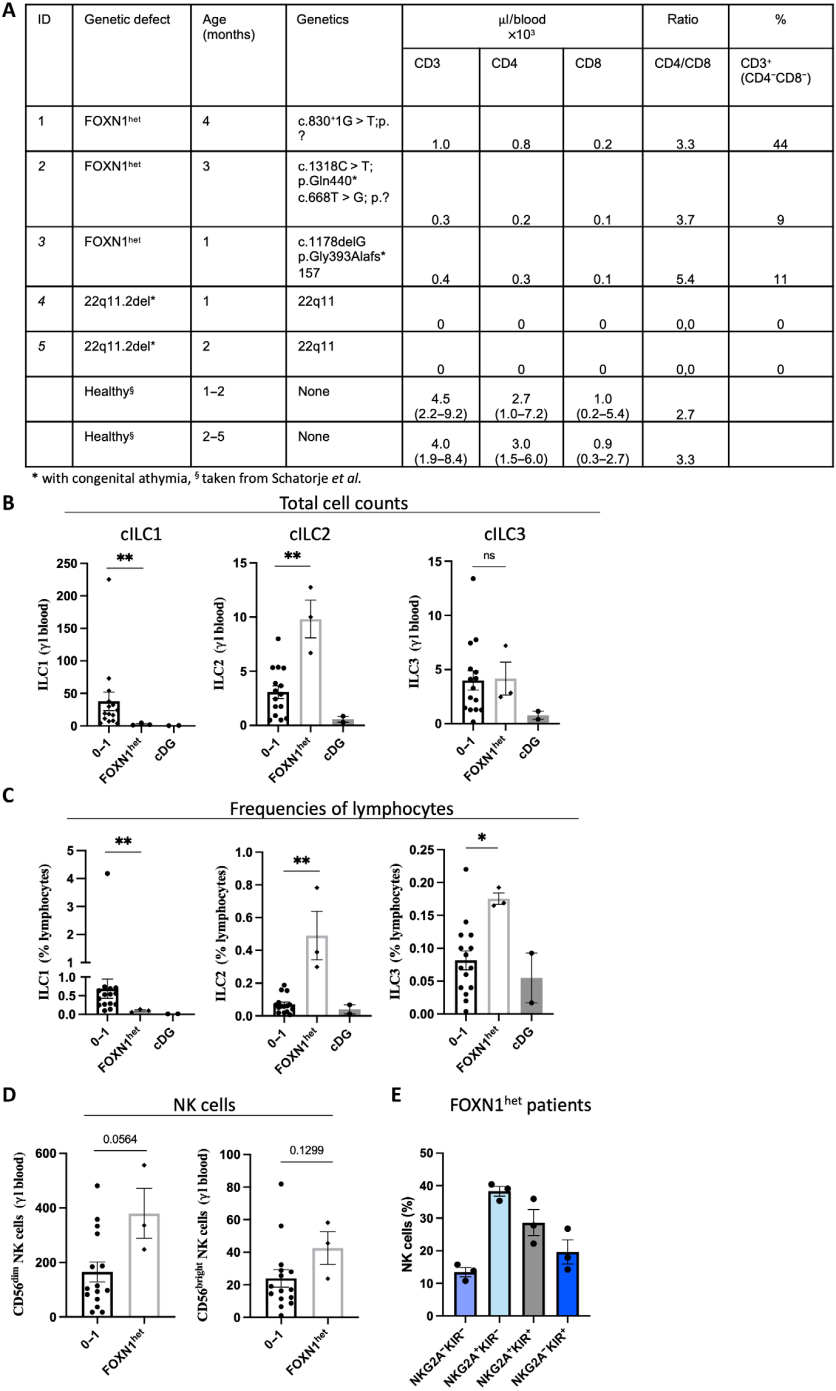
cILC1s are significantly decreased in patients with known inborn thymic defects. Blood from pediatric patients with *FOXN1^het^* (*n* = 3) and 22q11.2del with congenital athymia, also known as cDG (*n* = 2) was analyzed for the abundance of cILC1, cILC2, cILC3, and NK cells and compared to published reference values of cILC1-3 of age-matched children between the age 0 and 1 (*23*). (**A**) Summary table that categorizes the patients with respect to the genetic defects, age, and laboratory parameters. Bar graphs showing (**B**) total cell count (per microliter of blood) and (**C**) frequencies for cILC1s, cILC2s, and cILC3s in *FOXN1*^het^ (*n* = 3) and patients with cDG (*n* = 2) compared to age-matched controls (*n* = 15). (**D**) Bar graphs showing total cell counts (per microliter of blood) for CD56^dim^ and CD56^bright^ NK cells from patients with *FOXN1*^*he*t^ (*n* = 3) in comparison to age-matched healthy controls (*n* = 15). (**E**) Bar graphs showing the frequencies of NK cell subsets based on NKG2A and KIR expression in patients with *FOXN1*^het^ (*n* = 3). The height of the bars represents the means ± SEM. Levels of significance were calculated between the healthy cohort and the patients with *FOXN1^het^* a Wilcoxon test. Because of the small sample size, no statistical tests could be calculated with the patients with cDG, *P < 0.05, **P < 0.01.

So far, we could show that thyILC1s have NKPs potential with a bias toward differentiation into KIR-expressing NK cells. As this is reminiscent of the NKP properties of cILC1s (*7*), we wanted to further explore the developmental relationship between these two subsets. We reasoned that cILC1s would be decreased in patients with thymic defects if thyILC1s would be their direct progenitors. To this end, we took the opportunity to analyze patients with diagnosed FOXN1^heterogenous^ (*FOXN1*^het^) mutations (*n* = 3), a rare gene defect compromising thymic architecture due to a lack of functional TECs that leads to thymic hypoplasia (Fig. 8A). These patients were diagnosed after birth within the TCR excision circle (TREC) newborn screening, which has been implemented in 2019 to detect patients with severe T cell deficiency. Since FOXN1 is not expressed in cells of the hematopoietic system, it enables a selective view on the role of the thymic environment on the development of thymocytes and ILCs. Patients with *FOXN1*^het^ revealed a profound lack of cILC1s in terms of absolute cell number and in percentage of lymphocytes compared to reference values of age-matched healthy controls (Fig. 8, B and C) (*23*). In contrast, cILC2s were significantly increased in terms of absolute cell numbers and frequency, whereas cILC3s had unchanged absolute cell numbers but increased cell frequencies. Thus, cILC1s were selectively and significantly reduced in cell counts and frequencies in patients with *FOXN1*^het^, suggesting a tight dependency of cILC1 development on a functional thymus.

In contrast, patients with *FOXN1^het^* showed elevated CD56^dim^ and CD56^bright^ NK cell counts compared to age-matched controls (Fig. 8D). Notably, they also had sizeable subsets of all four KIR/NKG2A subsets including a KIR^+^NKG2A^−^ NK cell subset (mean: 19.64%) (Fig. 8E). Unfortunately, reference values of the respective KIR/NKG2A subsets in healthy controls of this age group (<1 years) were not available for comparison. Nonetheless, the data show that patients with *FOXN1^het^*, despite the almost complete absence of cILC1s, were still able to develop KIR^+^NKG2A^−^ NK cells.

We further analyzed two patients with complete DiGeorge syndrome (cDG), a disease characterized by a heterozygous microdeletion on 22q11.2del and congenital athymia. Similar to the patients with *FOXN1*^het^, cILC1s were strongly reduced in patients with cDG in terms of cell numbers and frequencies. However, in contrast to patients with *FOXN1*^het^, all three ILC subsets were strongly reduced in patients with cDG, with cILC2s showing an almost complete lack and cILC3s also being strongly reduced (Fig. 8, B and C). Of note, similar to our observation in the patients with FOXN1^het^, we observed elevated CD56^dim^ and CD56^bright^ NK cell frequencies and expression of KIR^+^ NK cells with and without NKG2A coexpression in patients with cDG (fig. S8). The results are again compatible with a role of the thymus in ILC development, but the almost complete lack of all ILCs suggests a block in development from a common ILC progenitor in patients with cDG.

## DISCUSSION

In the present study, we performed a first comprehensive survey of ILC subsets in the human thymus. thyILC1s, defined as lin^−^CD34^−^CD117^−^CRTH2^−^CD5^+^CD127^+^, were found to be the most abundant ILC population in thymus, also outnumbering NK cells. We demonstrate that thyILC1s are on the one hand potent producers of IFN-γ, constituting a key effector feature of tissue ILC1s (*3*, *48*), and on the other hand constitute an NKP with the ability to efficiently differentiate into KIR^+^NKG2A^−^ NK cells, a highly functional NK cell subset that so far could not be efficiently generated from previously defined NKPs. Last, in patients with a defect in thymus function due to heterozygous FOXN1 mutations, cILC1s were absent, suggesting that thyILC1s are the progenitors of cILC1s.

thyILC1s are distinguished from previously defined NKPs by the lack of CD34 and CD117 expression. Classical NKPs are characterized by expression of either CD34 and/or CD117 and are found in BM (phenotype: CD34^+^), SLNs (phenotype: CD34^+^CD117^+^, CD34^−^CD117^+^, and CD117^+^CD56^bright^), or the circulation (phenotype: CD117^+^CD56^bright^). In addition, bipotential CD34^+^ T/NKPs were described in the thymus in humans and mice (*29*, *52*, *53*). However, none of these progenitors were able to efficiently differentiate in vitro into KIR^+^NKG2A^−^ NK cells (*16*, *54*–*57*). Studies in clinical hematopoietic stem cell transplantation revealed that NK cell reconstitution is characterized by an early wave of CD56^bright^ NKG2A^+^ NK cells followed by a rather slow recovering of KIR^+^ and KIR^+^NKG2A^−^ NK cells (*58*, *59*). It is thus widely assumed that classical early CD34^+^ NKPs in BM and more advanced CD34^−^CD117^+^ NKPs residing in SLNs have the intrinsic ability to differentiate into KIR^+^NKG2A^−^ NK cells but that the in vitro conditions do not adequately support this late step of differentiation. Meanwhile, we show that thyILC1s differentiate into KIR^+^NKG2A^−^ NK cells with much higher efficiency than immature thyNK cells (this study), early CD34^+^NKPs (*16*), or CD56^bright^ NK cells from the circulation (*7*), using comparable in vitro conditions, suggesting that the intrinsic potential to mature to late developmental stages, such as the KIR^+^NKG2A^−^ stage, is different for different types of NKPs.

The molecular basis for the increased competency of thyILC1 to progress toward the KIR^+^NKG2A^−^ NK cell stage is currently unknown, but the thymus environment might play an important role to imprint thyILC1s for progression along this developmental pathway. The induction of KIR expression during NK cell differentiation is dependent on NOTCH signaling (*7*, *16*, *60*–*62*), and we had previously shown that cILC1 cells require OP9 stroma cells expressing the NOTCH ligand DLL1 to proceed to the KIR^+^NKG2A^−^ stage, whereas OP9 without DLL1 did not progress beyond the KIR^+^NKG2A^+^ stage (*7*). Notably, TECs are known to depend as well as express high levels of NOTCH ligands (*63*, *64*), and thyILC1s were shown here to strongly express NOTCH. Of note, strong NOTCH signaling was previously shown to prime human thymic CD34^+^CD1a^+/−^ DNs toward an ILC2 fate at the expense of a T cell fate (*27*). In particular, NOTCH3 stands out as it is strongly expressed in thyILC1s (and DN and ISP thymocytes), but is absent in thyNK cells. It is thus possible that NOTCH3 constitutes a thymus-specific feature of thyILC1s, promoting a molecular imprinting that enables the generation of advanced NK cell stages such as KIR-only NK cells.

Transcriptomic analysis by scRNA-seq revealed that thyILC1s are most closely related to DN-stage thymocytes and similar to the latter are characterized by high expression of *PTCRA* (preTCRα), *RAG1*, *RAG2*, *NOTCH1*, and *NOTCH3* as well as low-level transcription for *TRBV* and *TRAV* genes. However, in contrast to DNs, thyILC1s lacked CD34 expression and were unable to proceed to the CD4^+^ ISP stage when subjected to T cell differentiation conditions in vitro. Other groups detected residual *TRBV* and *TRAV* transcripts in human tILC1s, cILC1s, and cILC2s (*41*, *42*, *65*), already prompting considerations that these ILCs might have originated in the thymus (*66*, *67*). The block in differentiation along the T cell lineage in thyILC1s was associated with the failure to express productively rearranged TCRVβ chains as documented by the lack of intracellular TCR constant β 1 chain protein. Whereas, as expected, about 50% of ISPs exhibited intracellular expression of the TCRCβ1 protein (supposedly the other 50% expressing the TCRCβ2 chain), the TCRCβ1 chain was undetectable in thyILC1s. Notably, the default fate of thymocytes, which have down-regulated CD34 and failed to express the preTCR complex, is apoptosis (*33*, *35*). Obviously, this seems not to be the case for thyILC1s, which do not exhibit apoptosis-related transcriptional signatures in scRNA-seq analysis and have gained potent ILC1-like effector functions. Together, these data are compatible with a model where thyILC1s originate from DNs that did not pass the β-selection checkpoint toward T cell commitment but accessed an alternative differentiation pathway toward thyILC1s. This pathway involves rescue from apoptosis and as our data suggest commitment to the NK cell lineage. It needs to be mentioned that we also generate CD117^+^NKp44^+^ cells within our cultures (fig. S3B), which might either be stage 3 NKPs (*55*) or ILC3s (*68*). Therefore, we cannot completely rule out that thyILC1s might be a precursor also for other innate cells apart from NK cells. This needs to be further investigated in follow-up studies.

The potential molecular mechanisms that might have protected these progenitors from apoptosis and leading to an alternative pathway toward thyILC1s are so far unknown and need further investigation. Of note, previous studies have already shown that not all DNs commit to a T cell fate. In line with our observations, thymic CD34^+^ DNs were previously shown to have NK cell differentiation potential (*25*, *69*). Furthermore, rearranged TCRγ genes have been identified within NK cells (*70*) further suggesting NK cell development from the DN stage. These studies suggest the presence of a bipotent T/NK precursor in the thymus (*33*, *71*). The decision between a T or NK cell fate was suggested to be induced by intrinsic regulators such as the TFs ID3 and BCL11b, promoting or blocking NK cell development (*25*, *69*, *72*). In the case of ID3, its expression and also the *ID3/ID2* ratio increased from thyILC1s toward CD4^+^ ISPs, arguing against a role of ID3 as a switch factor toward an NK cell fate. Similarly, although BCL11b expression was suggested to block NK cell development in a knockout mouse model (*73*), our data show strong expression of BCL11b in thyILC1s (fig. S5B), again arguing against a prominent role as negative regulator in this process. On the contrary, a recent study showed the importance for BCL11b expression during human NK cell development (*72*).This could suggest a potential involvement of BCL11b during fate decision in humans, which seems to be differently regulated in mice. scRNA-seq also revealed that thyILC1s expressed the TF *SOX4* at higher levels than all other thymic subsets. SOX4 is an essential developmental TF that also plays key roles in myeloid and lymphoid hematopoiesis (*74*). Further to this, it was recently shown that SOX4 plays an important role in regulating NKT cell and T- to NK-like T cell transition (*75*, *76*). SOX4 expression might thus indeed be one factor influencing the functional switch from T cell toward NK cell commitment at the DN stage, but the regulated target genes remain to be defined.

KIR^+^NKG2A^−^ NK cells are mainly found in the circulation where they typically make up >30% of NK cells (*9*), whereas they are rather rare in the thymus (<5% of thyNK cells) (*36*). It appears thus unlikely that thyILC1s differentiate into KIR^+^NKG2A^−^ NK cells on site but rather migrate into the circulation and differentiate into cILC1s. This maturation process involves shutting down the TCR somatic recombination machinery including RAG1, RAG2, and PTCRA, but an up-regulation of surface receptors such as CD161, and eventually down-regulation of CD5 expression (*7*). A progenitor/descendant relationship between thyILC1s and cILC1s is apparent not only because of the phenotypical, molecular, and functional similarities between both subsets but also suggested by the analysis of patients with congenital thymic defects. We could show that heterozygous loss-of-function FOXN1 mutations in three pediatric patients not only led to T cell lymphopenia but also a lack of cILC1s, whereas other innate lymphocytes either remained unchanged (cILC3) or even increased (cILC2 and NK cells). FOXN1 is a master regulator for TEC development (*77*), and its expression is largely confined to TECs and skin cells, leaving other sites of hematopoiesis such as the liver, BM, and lymphatic tissues unaffected. This establishes a close connection between thymic hematopoiesis and the development of cILC1s and suggests that the cILC1 deficiency in patients with FOXN1^het^ is due to the lack of thyILC1s, which further suggests that thyIC1s are a nonredundant cILC1 progenitor.

Notably, we observed an almost complete absence of all ILC subsets in athymic patients with cDG. Although this could indicate a role for the thymus for the development of all cILC subsets, this has to be interpreted with caution since patients with cDG have a 22q11.2del microdeletion involving >30 different genes, leading to complex pathology involving cardiac abnormalities and hypoparathyroidism among others. It is thus difficult to determine whether the lack of all ILC subsets is specifically due to thymic aplasia (*78*). On the other hand, a correlation between murine cILC precursors with thymic functionality was observed in scRNA-seq (*79*), suggesting that the thymus might not only be important for cILC1s but also for other cILCs.

Although all patients with thymic defects were deficient for cILC1s, they had NK cells, including KIR^+^NKG2A^−^ NK cells, suggesting that they could compensate for the lack of cILC1-derived NK cells and that imprinting for KIR expression can take place in alternative niches within the human body. The presence of KIR^+^NKG2A^−^ NK cells in these patients could be due to the existence of other yet unidentified NKPs biased toward generating KIR^+^ NK cells or alternatively that the known CD117^+^ NKPs (CD34^+^, CD56^bright^ NK cells) might need different, so far unknown, in vitro stimuli or in vivo priming compared to thyILC1s/cILC1s. The observation further strengthens the idea of a branched NK cell developmental pathway, where different NKPs generate the diverse pool of NK cell populations seen in vivo, instead of a linear model, where one precursor precedes the next. Notably, also T cells of patients with FOXN1^het^ typically recover over time without further intervention (*80*). A similar phenomenon was recently also described in a study investigating patients with biallelic pre-TCRα deficiency, which suggested a noncanonical T cell differentiation pathway that circumvents the thymic β-selection checkpoint (*81*).

In summary, thyILC1s constitute a thymic NKP that has unique phenotypical, molecular, and functional properties compared to previously defined NKPs in the BM, liver, or secondary lymphoid tissues. First of all, thyILC1s are characterized by a DN-like transcriptional signature including key genes of the TCR recombination machinery but differ from these by the absence of CD34 expression and lack of T cell differentiation potential. We show that both DN thymocytes and thyILC1s have NK cell differentiation potential, but only thyILC1s are biased toward expression of KIR, in particular KIR^+^NKG2A^−^ NK cells, whereas DN thymocytes primarily develop into NKG2A^+^ NK cells. The selective deficiency in cILC1s in patients with *FOXN1^het^* establishes thyILC1s as prime candidate for being the progenitor of cILC1s, a circulating NKP with similar properties toward differentiation into KIR^+^NKG2A^−^ NK cells. However, our data further suggest that thyILC1s/cILC1s might not be the only NKP with the potential to generate KIR-only, as patients with FOXN1^het^ do not show a selective loss of KIR^+^ NK cells. These KIR-only NK cells are the basis for clonally diversified NK cell repertoires and majorly contribute to the functional diversity of circulating human NK cells. The ability to generate KIR^+^NKG2A^−^ NK cells from thyILC1s and cILC1s in vitro provides novel opportunities to study NK cell education by cognate HLA class I ligands, a process that is so far poorly understood on the molecular level. Furthermore, it opens novel possibilities to establish protocols for the selective expansion of KIR-only NK cells, which are anticipated to be highly versatile for the detection and eradication of tumor cells with aberrant HLA class I expression and that are unaffected by tumor-mediated inhibition through the HLA-E-NKG2A axis. Last, the modification of in vitro–generated polyclonal KIR-only NK cells with chimeric antigen receptors (CARs) could emerge as an interesting previously unknown option for tumor-specific NK cell immunotherapy besides conventional CAR NK cells that are majorly KIR^−^NKG2A^+^ (*82*).

## MATERIALS AND METHODS

### Human samples and ethics statement

Postnatal thymic (PNT) samples were collected from immunologically healthy children (age between 2 days and 46 weeks), who underwent cardiac surgery (Herzzentrum Duisburg, Evangelisches Klinikum Niederrhein). The protocol was accepted by the Institutional Review Board at the University of Düsseldorf (study number 2020_1242). The organs were placed in cold (4°C) RPMI medium 1640 + GlutaMAX (Gibco) immediately after removal and processed either on the day of the surgery or on the next day. Blood samples of pediatric patients diagnosed with *FOXN1*-heterozygous mutations and from patients with cDG were collected from the Department of Pediatric Oncology, Hematology and Clinical Immunology, University Hospital Duesseldorf (study number 2020-1201_1, registered at the German Clinical Trials Register: DRKS00032712). The patients were diagnosed as newborns within the TREC newborn screening (*83*). Informed consent was obtained from the legal guardians.

### Isolation of thymocytes from PNT

Thymocytes were isolated from PNTs by cutting the organ into small pieces with dissecting scissors and covering them with ice-cold 1× Dulbecco’s phosphate-buffered saline (DPBS) (Gibco) and staining buffer [DPBS + 0.5% bovine serum albumin (Roth) + 2 mM Versene (EDTA) (Gibco) (2:1)] in a sterile six-well plate. The thymocytes were squeezed out of the organ pieces into the liquid with a sterile stamp. Afterward, thymocytes were harvested with a pipette and transferred into a sterile 50-ml tube. Squeezing and collection of thymocytes was performed multiple times until no further cells could be squeezed out of the tissue pieces. The collected cells were either used for subsequent experiments immediately or frozen down in RPMI medium 1640 + GlutaMAX (Gibco) with 50% fetal calf serum (FCS, Merck) and 10% dimethyl sulfoxide (Roth) for future usage [Protocol from (*47*)].

### Isolation of MNCs from umbilical CB or PB

From each CB sample, an undiluted aliquot of whole blood, and for each pediatric PB sample, an aliquot of whole blood was diluted with 1:10 1× PBS for whole-blood cell count (Cell Dyn 3500R, Abbot Laboratories). CB (1:1) and PB of pediatric patients (1:5) were diluted with sterile 1× PBS (Gibco) and mononuclear cells (MNCs) were isolated by density gradient centrifugation (Lymphocyte separation Medium 1077, PromoCell). The remaining erythrocytes were lysed with ice-cold ammonium chloride solution (pH = 7.4, University Clinic Düsseldorf) and washed two times afterward. MNCs were counted and cryopreserved or directly used for further analyses.

### Cell cultivation and passaging

The murine stroma cell line OP9-DL1 was provided by J. C. Zúñiga-Pflücker, University of Toronto, and was cultivated in Dulbecco’s modified Eagle’s medium (DMEM) low-glucose (1 g/liter) + Glutamax (Gibco) with 1% penicillin-streptavidin (Gibco) and 10% FCS (Merck). The cells were regularly screened by flow cytometry for DL1 expression (the cells were green fluorescent protein positive). Good manufacturing practice–grade MSCs were obtained as previously described (*16*). MSCs were cultured in Dulbecco’s high-grade glucose medium (Gibco) with 10% platelet lysate and 1% penicillin-streptomycin in T175 culture flasks. Both cell lines were passaged using 1× Trypsin (Gibco) at a density of approximately 85%. The major histocompatibility complex class I negative suspension cell line K562 was cultured in DMEM high-glucose (4.5 g/liter) (Gibco) medium + 10% FCS (Merck). All cells were cultivated at 37°C and 5% CO_2_ and tested negative for mycoplasma.

### Flow cytometry analysis

PNT samples were either stained immediately following cell isolation or if frozen, thymocytes were first separated via density gradient centrifugation (lymphocyte separation medium 1077, PromoCell) and subsequently stained. For extracellular staining of thyILCs, a Lineage cocktail (described in the section: thyILC1s are the predominant innate lymphocyte subset in human thymus) was used. For intracellular staining of cytokines, the cells were permeabilized subsequent to extracellular staining using the intracellular staining kit (BioLegend) according to the supplier’s protocol. For intranuclear staining for the detection of expression of TFs, the intranuclear Foxp3/TF staining kit (Invitrogen, eBioscience) was used for permeabilization after extracellular staining (list of antibodies used in table S2).

### Stimulation of PNT ILCs and NK cells

To analyze the functionality of thyNK cells and ILC1, thymocytes were either stimulated immediately after isolation from PNT or frozen and stimulated after density gradient centrifugation. For stimulation, the thymocytes were seeded in a 96-U bottom plate in RPMI medium 1640 + GlutaMAX (Gibco) + 10% FCS (Merck) (STD Media) with IL-12 (5 ng/ml) and IL-18 (50 ng/ml). After a 1-hour incubation step (37°C, 5% CO_2_), Monensin (final concentration 1×, BioLegend) was added to each well and incubated for an additional 16 hours. Subsequently, the cells were harvested and stained.

### Cocultivation of OP9-DL1 with primary thyILC1s or NK cells

OP9-DL1 feeder cells (2000 to 5000) per well were transferred into a 96-well flat bottom tissue culture plate 1 to 2 days before cocultivation of bulk-sorted thymocytes. One or two days after seeding the feeder cells, PNT samples were sorted either from frozen samples or immediately after isolation. Frozen thymic samples were thawed, and thymocytes were isolated by density gradient centrifugation (lymphocyte separation medium 1077, PromoCell). First, CD3- and CD1a-expressing thymocytes were depleted using biotin-labeled antibodies against CD3 (clone: OKT3) and CD1a (clone HI149) and the MojoSort Streptavidin Nanobeads (both BioLegend) using the suppliers negative selection protocol as described previous (*7*). After depletion, the thymocytes were stained with a lineage cocktail of fluorescein isothiocyanate (FITC)–coupled antibodies: anti-CD3 (UCTH1), anti-CD11c (3.9), anti-CD14 (HCD14), anti-CD19 (HIB19), anti-CD123 (6H6), anti-CD235a (HI264), anti-TCRαβ (IP26), anti-TCRγδ (B1), anti-FcεR1α (AER-37) (all from BioLegend), anti-CD4 (13B8.2) and anti-CD8 (B9.11) (both Beckmann Coulter). In addition, the cells were stained for ILC inclusion marker CD127 phycoerythrin (PE)–Cy5 (clone: R34.34 Beckmann Coulter), NK cell marker CD94–allophycocyanin (APC) (clone: DX22, BioLegend), hematopoietic stem cell marker CD34-PE (clone: 581, BioLegend), as well as CD117-PE-Cy7 (clone: 104D2, BioLegend), and CRTH2-PE-Dazzle (clone: BM16, BioLegend) to distinguish between the ILC subsets. The stained PNT samples were used to flow cytometrically sort thyILC1s (lin^−^ CD127^+^ CD34^−^ CD94^−^ CD117^−^ CRTH2^−^) and NK cells (lin^−^ CD94^+^). For cocultivation of bulk-sorted populations, 500 to 2500 ILC1 or NK cells were seeded on feeder cells either in 24-well plates (MSCs) or 96-well plates (OP9-DL1) in “NK2” media [66.6% DMEM high-glucose (4.5 g/liter) (Gibco), 33.3% HAM’s F12 Nutrient Mixture (Gibco), 10% human AB serum, 1% l-Glutamine (Gibco), 1% penicillin-streptomycin (stock: 10,000 U/ml, 10,000 μg/ml streptomycin) (Gibco), 50 μM ethanolamine (Sigma-Aldrich), sodium selenite (50 μg/liter, Sigma-Aldrich), 24 μM 2-Mercaptoethanol (Gibco), IL-2 (500 U/ml), IL-7 (10 ng/ml), SCF (10 ng/ml), Flt3-L (5 ng/ml), and IL-15 (5 ng/ml) (all cytokines from Miltenyi Biotec)]. For bulk-sorted cocultivation, the media were changed every 3 to 5 days by exchanging half of the media with fresh NK2 media. The cells were analyzed after 14 to 15 days of cocultivation.

### CD107 degranulation assay

The in vitro–generated effector cells (section “Cocultivation of OP9-DL1 with primary thymic ILC1s or NK cells”) were harvested from the coculture, filtered through a 30 μM strainer, and resuspended in media containing IL-2 (200 U/ml). For the CD107 degranulation assay, effector and K562 target cells were seeded in a 96 U bottom well in a 1:1 ratio with an anti-CD107 BV510 antibody (1:200 dilution, H4A3, BioLegend). After 1 hour of incubation at 37°C and 5% CO_2_, Brefeldin A and Monensin (final conc. 1×, both BioLegend) were added. After an additional 4 hours of incubation, the cells were harvested, stained and analyzed via flow cytometry. In addition, for each sample, a nontarget control was included to analyze spontaneous CD107 expression.

### Cytotoxicity assay

To evaluate the ability of the generated cells to specifically lyse target cells, a CFSE/PI-based cytotoxicity assay was performed. Therefore 1 × 10^7^ K562 target cells were labeled with 5 μM CFSE in 200 μl of PBS for 10 min at 37°C and 5% CO_2_. Subsequently, the cells were washed twice using PBS + 20% FCS and then added in a ratio of 1:1 to the in vitro–generated effector cells (section “Cocultivation of OP9-DL1 with primary thymic ILC1s or NK cells”) in a 96-well round bottom plate in media containing IL-2 (200 U/ml). The cells were incubated for 5 hours 37°C and 5% CO_2_. Shortly before flow cytometric analyses, 1 μl of PI (BioLegend) was added. Spontaneous target cell death was determined in wells containing target cells alone. Specific lysis was calculated by

$$\begin{array}{c} \%\text{Specific lysis}=\frac{\left(\%{\text{CFSE}}^{+}{\mathrm{PI}}^{+}\text{with effector cells}-\%{\text{CFSE}}^{+}{\mathrm{PI}}^{+}\;\text{without effector cells}\right)}{\left(100-\%{\text{CFSE}}^{+}{\mathrm{PI}}^{+}\;\text{without effector cells}\right)}\times 100 \end{array}$$

### Bulk RNA-seq and analyses

For isolation of thyILC1s, frozen thymocytes were thawed, separated by density gradient centrifugation, magnetically depleted, and flow cytometrically sorted, as described for the coculture experiments (fig. S4, A to C, for sort strategy). For sorting of ISPs, DN3, and CD4^+^ T cells, thymocytes were immediately stained after the density gradient centrifugation step with an FITC-coupled lineage cocktail [anti-CD11c (3.9), anti-CD14 (HCD14), anti-CD19 (HIB19), anti-CD94 (DX22), anti-CD123 (6H6), anti-CD235a (HI264), anti-TCRγδ (B1), and anti-FcεR1α (AER-37) (all from BioLegend)], anti-CD34 PE (clone: 581, BioLegend), anti-CD4 PE-Cy7 (OKT4, BioLegend), anti-CD1a APC (HI149, BioLegend), anti-CD8 AF700 (RPA-T8, BioLegend), and anti-CD3 APC-Cy7 (UCTH1, BioLegend). DN3s were defined as lin^−^ CD34^+^CD1a^+^CD3^−^CD4^−^CD8^−^, ISPs as lin^−^ CD34^−^CD1a^+^CD4^+^CD3^−^CD8^−^ (fig. S4, A to C), and CD4^+^ T-cells as lin^−^ CD1a^−^CD3^+^CD4^+^CD8^−^. For each population, 1 to 2 × 10^4^ cells were used. RNA extraction, library preparation, and Next Generation Sequencing (NGS) with the Illumina Truseq RNA preparation kit was performed in Göttingen (G. Salinas-Riester, NGS-Integrative Genomics Core Unit, University Medical Center, Göttingen) according to the manufacturer’s protocols. mRNA sequencing of the libraries was performed with an Illumina HiSeq4000 sequencer [paired-read 2 × 50 base pair (bp)]. Sequence reads were mapped using the computational infrastructure at the Heinrich-Heine University Düsseldorf (Centre for Information and Media Technology at Heinrich Heine University Düsseldorf). Forward and reverse reads were mapped against the human genome (hg38, Homo.sapiens.GRCh38.dna.primary_assembly.fa) with the gtf file `Homo.sapiens.GRCh38.84.chr.gtf` using the nf-core/rnaseq pipeline (*84*) (version 3.8, Profile Singularity) via Nextflow (version 23.10.0) (*85*). Embedded softwares contained bedtools (version 2.30.0), python (version 3.9.5), star (version 2.7.10a), salmon (version 1.5.2), and samtools (version 1.15.1).

RNA-seq analyses was performed with R (version 4.2.1) (*86*) and R studio (versions 2022.07.2) (*87*) with the R packages tidyverse (tidyverse_2.0.0) (*88*) containing dplyr (dplyr_1.1.2), and readr (readr_2.1.4) as well as useful (useful_1.2.6). Analysis of differential gene expression, normalization of read counts, and PCA were performed with the R package DESeq2 (DESeq2_1.36.0) (*89*). Heatmaps and volcano plots were generated using the R packages pheatmap (pheatmap_1.0.12) and EnhancedVolcano (EnhancedVolcano_1.14.0) (*90*). For the heatmaps, the top 100 differentially expressed genes were taken with a cutoff of *P*-adjust <0.05. To change color patterns, the R packages RColorBrewer (RColorBrewer_1.1-3) and ggplot2 (ggplot2_3.4.2) (*91*) were used.

### scRNA-seq using BD rhapsody

Frozen thymocytes were thawed, separated by density gradient centrifugation, and T cells magnetically depleted using an anti-CD3 biotin-coupled antibodies (clone: OKT3, BioLegend). The remaining cells were stained with an FITC-coupled lineage cocktail containing anti-CD11c (3.9), anti-CD14 (HCD14), anti-CD19 (HIB19), anti-CD123 (6H6), anti-CD235a (HI264), anti-TCRγδ (B1), and anti-FcεR1α (AER-37) (all from BioLegend), anti-CD34 PE (clone: 581, BioLegend), anti-CD127 PE-Cy5 (R34.34, Beckmann Coulter), anti-CD94 PE-Cy7 (DX22, BioLegend), anti-CD4 APC (OKT4, BioLegend), anti-CD8 AF700 (RPA-T8, BioLegend), and anti-CD3 APC-Cy7 (UCTH1, BioLegend). Three PNTs were used for scRNA-seq using the BD Rhapsody system, and for each thymus, three populations were sorted for subsequent multiplexing: Lin^−^ CD3^−^ CD4^+^ and/or CD8^+^ to include all ISPs and early DPs (population 1), Lin^−^ CD3- CD4^−^ CD8^−^ CD34^+^ to include DN stages 1 to 3 (population 2), and lin^−^ CD3^−^ CD4^−^ CD8^−^ CD34^−^ CD94^+^ and/ or CD127^+^ to include NK cells and ILCs (population 3) (fig. S4D). For populations 1 and 2 only one sample tag each was used. Population 3 was separated into two sample tags per donor to amplify the yield. After the multiplexing step, equal amounts of cells per sample tag were pooled. The pooled samples were stained with the BD AbSeq Immune Discovery Panel (table S1) plus additional AbSeq antibodies [anti-CD34 (clone: 581), anti-CD117 (clone: YB5.B8), anti-CD1a (clone: HI149), anti-CD94 (clone: HP-3D9), CD336 (NKp44, clone: p44-8), and CD294 (CRTH2, clone: BM16) (all from BD)]. The Rhapsody device was loaded with a total of 60,000 multiplexed and AbSeq-stained thymocytes. The following single-cell capture and cDNA synthesis as well as exonuclease treatment was performed according to the manufacturer’s instructions and published protocol (*92*). After these steps, the cDNA sample was stored at 4°C for the library preparation and NGS. Library preparation and sequencing was performed at PRECISE, DZNE, Bonn (NovaSeq_S4, 200 bp. v1.5). The BD Rhapsody Sequence Analyses Pipeline (https://bd-rhapsody-bioinfo-docs.genomics.bd.com/top_introduction.html) was used with whole-transcriptome analyses and AbSeq Oligo labeling.

### scRNA-seq analyses

Seurat (version_4.3.0) was used for scRNA-seq analyses (*93*, *94*) with R (version 4.2.1) (*86*) and R studio (versions 2022.07.2) (*87*). After quality analyses with Seurat, a total of 9162 cells passed initial quality control for prefiltering, and 8842 cells remained post-filtering with a mitochondrial percentage (percent.mito) cutoff at 25, nFeature_RNA between 200 and 10,000, and n_count_RNA > 500. RunPCA was used with VariableFeatures, ElbowPlot, and JackStrawPlot to determine 10 dimensions (dims) for RunUMAP. Clustering was performed with FindNeighbors (10 dims) with a resolution of 0.5 for the scRNA-seq data of all three populations or a resolution of 0.3 to analyze population 3 (NK/ILCs) alone. Cluster markers were calculated with FindAllMarkers, and the top 10 differentially expressed genes are displayed. The R code used for the scRNA-seq analyses was generously provided by J. Schulte-Schrepping (*95*) and modified for the purpose of this study. The code for AbSeq data was taken from https://satijalab.org/seurat/archive/v3.2/multimodal_vignette.html.

### Statistical analysis

The produced data were analyzed with the GraphPad PRISM 10.1.2 software. The used statistical tests to evaluate the significance of the data are mentioned in the figure descriptions. The level of significance was labeled with a star (*) and was divided into four different levels of significance; *P* < 0.05 = *, *P* < 0.01 = **, *P* < 0.001 = ***, and *P* < 0.0001 = ****.
