# Supplementary material for: A human NK cell progenitor that originates in the thymus and generates KIR+NKG2A− NK cells
Source: Sci Adv. 2025 Aug 8;11(32):eadv9650. doi: 10.1126/sciadv.adv9650 (PMC12333686; doi:10.1126/sciadv.adv9650)
Supplement: Supplementary file 1 — Figs. S1 to S8 Tables S1 and S2 [file sciadv.adv9650_sm.pdf]

Science Advances
AAAS

Supplementary Materials for

A human NK cell progenitor that originates in the thymus and generates $KIR^{+}NKG2A^{-}$ NK cells

Julian Reiß *et al.*

Corresponding author: Sabrina B. Bennstein, sbennstein@ukaachen.de;
Markus Uhrberg, markus.uhrberg@med.uni-duesseldorf.de

Sci. Adv. **11**, eadv9650 (2025)
DOI: 10.1126/sciadv.adv9650

This PDF file includes:

Figs. S1 to S8
Tables S1 and S2

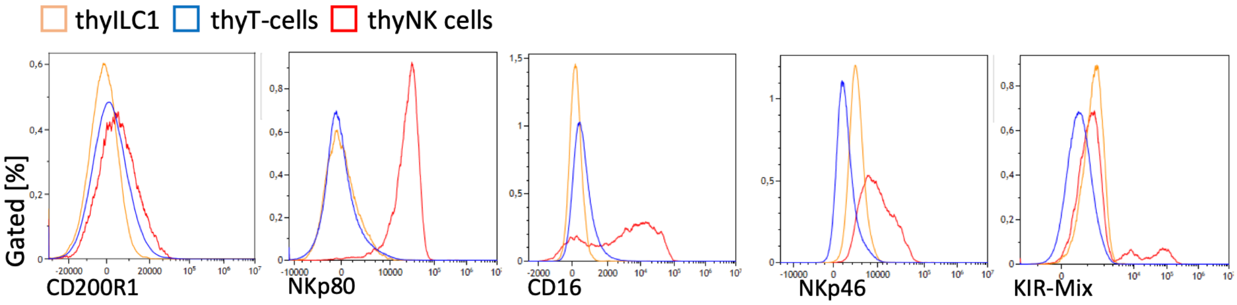

Supplementary Figure 1 | *Ex vivo* human thyILC1s lack expression of NK cell markers. To distinguish thymic ILC1s from NK cells and T cells, thymocytes isolated from PNT were stained and identified using the gating strategy shown in Figure 1. The representative histograms show the expression of CD200R1, NKp80, CD16, NKp46 and KIR-Mix of thyILC1s (yellow), thyNK cells (red) and thy$CD4^+$ T-cells (blue).

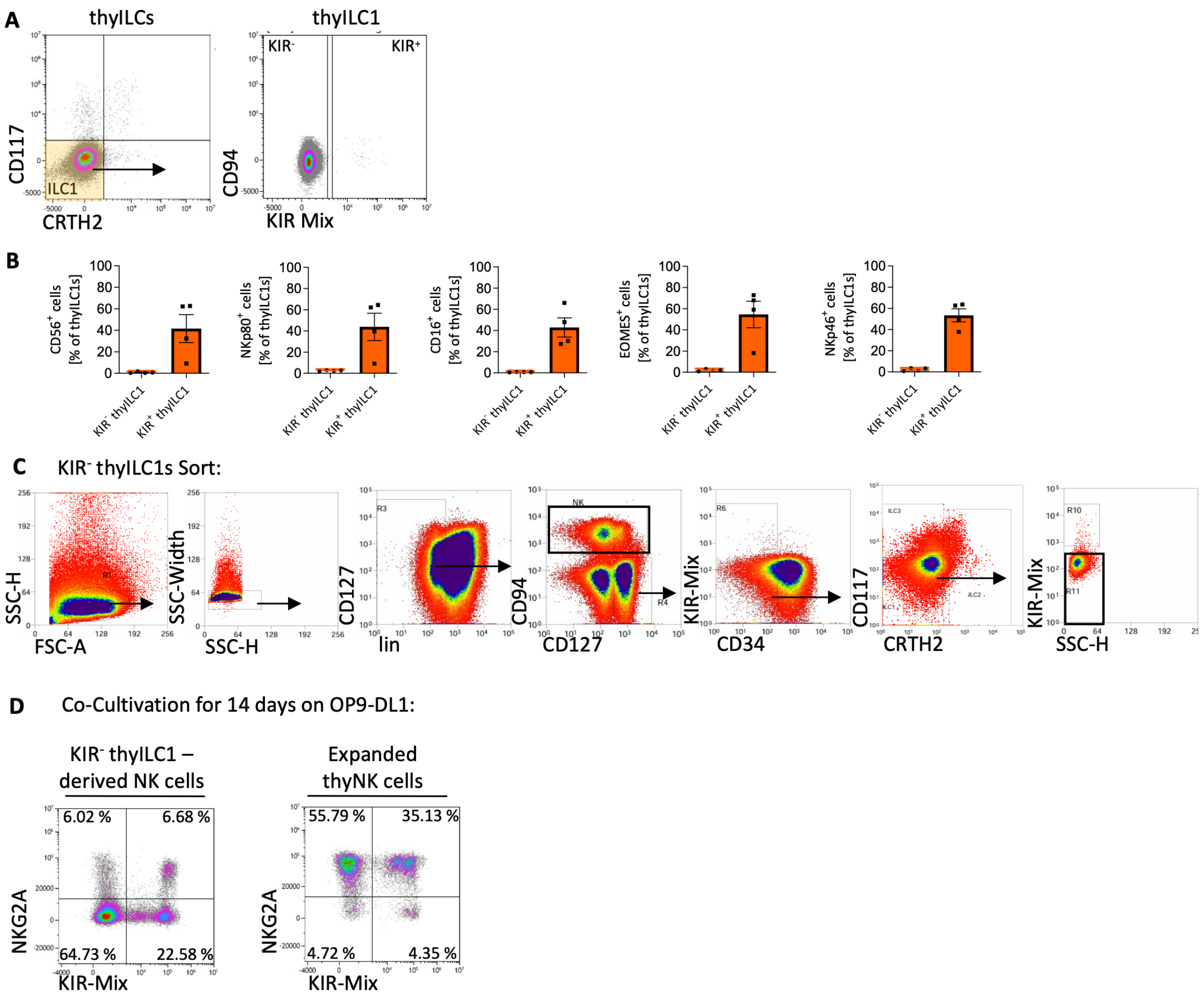

Supplementary Figure 2 | KIR$^-$ thyILC1 differentiate into mature KIR$^+$ NKG2A$^-$ NK cells.

To ensure that the generation of KIR$^+$ NKG2A$^-$ NK cells from thyILC1s (Figure 2) did not result solely from expansion of a minor KIR$^+$ cell fraction present in the thyILC1 population, KIR$^-$ thyILC1s were sorted and co-cultivated with OP9-DL1 feeder cells under NK cell differentiation supporting conditions (Section "Co-cultivation of OP9-DL1 with primary thymic ILC1s or NK cells"). **(A)** Representative Dot Plot showing the identification of KIR$^+$ cells present in the thyILC1 population. **(B)** Bar graphs comparing the expression of NK cell markers (CD56, NKp80, CD16, EOMES and NKp46) between KIR$^-$ and KIR$^+$ thyILC1s. **(C)** Exemplary sorting strategy to identify NK cells and KIR$^-$ thyILC1s. Prior to sorting CD3$^+$ cells were depleted. NK cells were identified as lin$^-$ (CD3, CD4, CD8, CD11c, CD14, CD19, CD123, CD235a, FCER1α, TCRαβ, TCRγδ) CD94$^+$. KIR$^-$ thyILC1s were defined as Lin$^-$ CD34$^-$ CD94$^-$ CD117$^-$ CRTH2$^-$ CD127$^+$ KIR$^-$. **(D)** Representative Dot Plots of NKG2A and KIR expression of the KIR$^-$ thyILC1 – derived NK cells and expanded NK cells after 14 days of co-cultivation (n = 4).

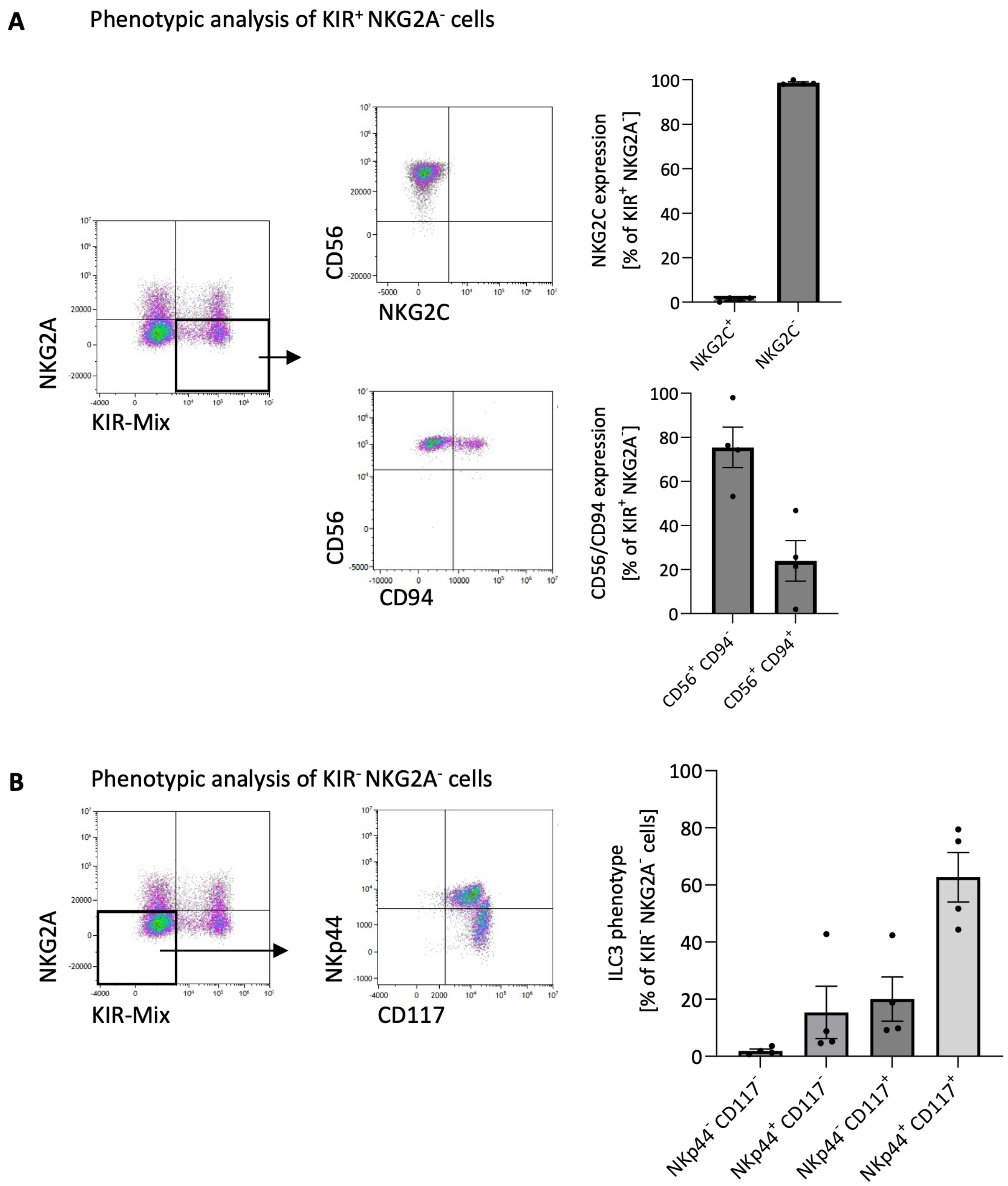

Supplementary Figure 3 | Phenotypic analysis of thyILC1-derived KIR$^+$ NKG2A$^-$ and KIR$^-$ NKG2A$^-$ cells.

To further characterize the NK cell subsets generated from thyILC1s, KIR$^+$ NKG2A$^-$ and KIR$^-$ NKG2A$^-$ were analyzed for additional surface marker (n = 4). **(A)** thyILC1-derived NK cells were further gated on KIR$^+$ NKG2A$^-$ and analyzed for NKG2C expression (upper dot plot and bar graph) as well as for CD56$^+$ CD94$^-$ and CD56$^+$ CD94$^+$ expression. **(B)** KIR$^-$ NKG2A$^-$ thyILC1-derived NK cells were analyzed for surface expression of CD117 and NKp44. The heights of the bars represent the mean ± SEM.

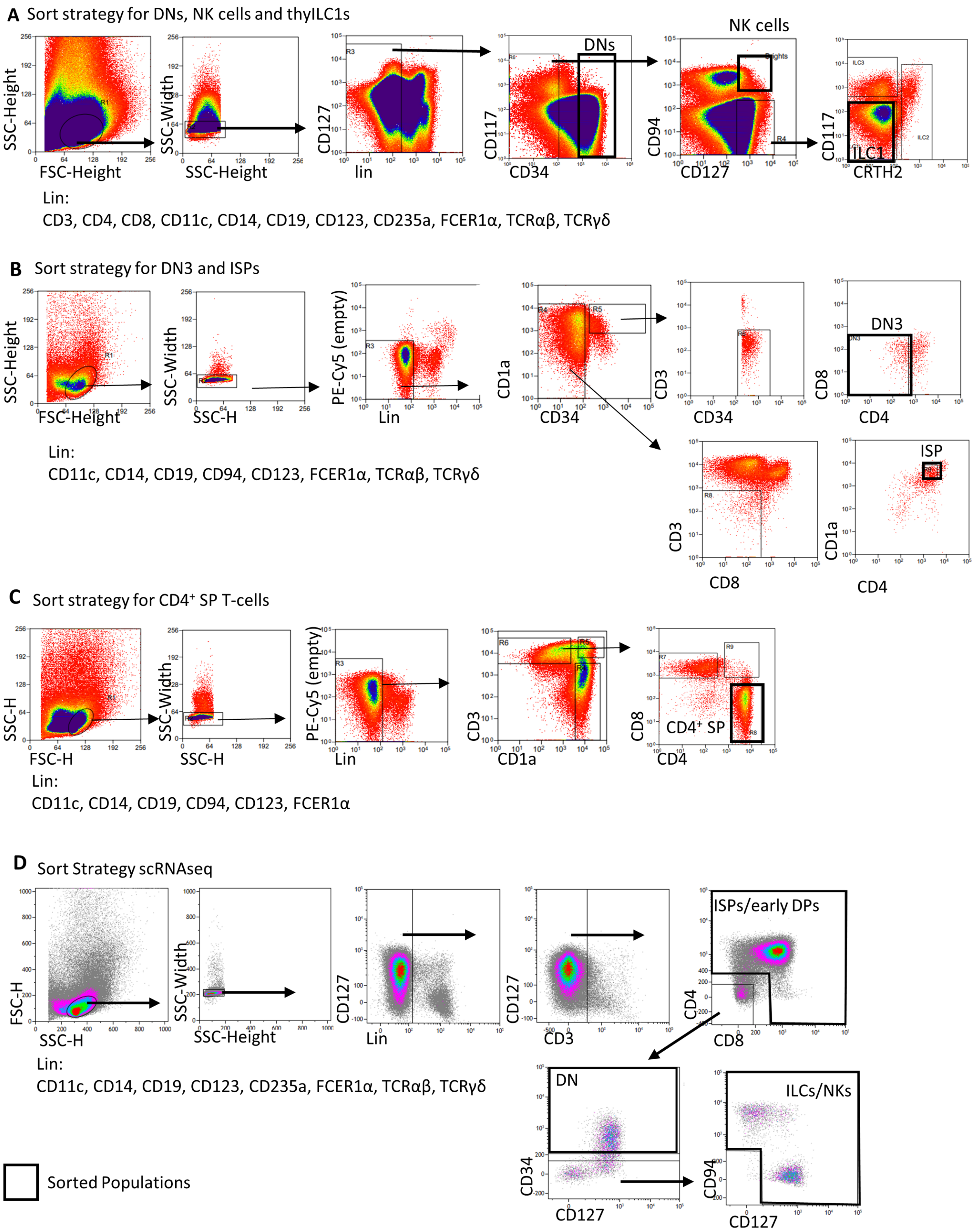

Supplementary Figure 4 | Sorting strategies for different thymic populations. Exemplary gating strategies for fluorescence activated cell sorting of different thymocyte populations. For each strategy, at first all thymocytes were identified and doublets were excluded prior excluding the lin^{+} cells. The different lineage cocktails are indicated beneath the associated sort strategy and at the end of this legend. For **(A)** and **(D)** the thymocytes were CD3 depleted prior staining for the FACS step. **(A)** Sort of DNs, NK cells and thyILC1s for *in vitro* differentiation and for bulk-RNAseq (for NK cells and thyILC1s). Lineage-negative cells were

additionally defined as $CD34^+$ DNs. Lin^- $CD34^-$ $CD94^+$ cells were further defined as NK cells. ThyILC1s were finally identified as Lin^- $CD34^-$ $CD94^-$ $CD117^-$ $CRTH2^-$ $CD127^+$. **(B)** For bulk-RNAseq of DN3s and ISPs lin^- were further subdivided upon their CD34 expression. $CD34^+$ cells were additionally gated on $CD1a^+$, $CD3^-$, $CD4^-$ and $CD8^-$ to identify DN3s. $CD34^-$ cells were further gated on $CD3^-$ and $CD8^-$ to lastly define lin^- $CD34^-$, $CD3^-$, $CD8^-$, $CD4^+$ and $CD1a^+$ ISPs. **(C)** To isolate $CD4^+$ Single Positive (SP) T-cells for bulk-RNAseq, lin^- were further gated on $CD3^+$, $CD1a^-$ to subsequently define $CD4^+$ Single Positive (SP) T-cells. **(D)** For scRNA-seq CD3 was separately excluded after lineage exclusion. Afterwards lin^- $CD3^-$ CD4 and/or CD8 expressing cells were sorted together as ISPs/ early DPs. Lin^- $CD3^-$ $CD4^-$ $CD8^-$ $CD34^+$ cells were sorted to obtain DNs. Finally, Lin^- $CD3^-$ $CD4^-$ $CD8^-$ $CD34^-$ CD94 and/or CD127 expressing cells were identified as Natural Killer (NK) cells / ILCs. The sorted populations are framed in black. The different Lineage cocktails included varying antibodies also indicated beneath the associated gating strategy: Lineage Cocktail **(A)**: CD11c, CD14, CD19, CD123, CD235a, FCER1α, TCRαβ, TCRγδ; Lineage Cocktail **(B)**: CD3, CD4, CD8, CD11c, CD14, CD19, CD123, CD235a, FCER1α, TCRαβ, TCRγδ; Lineage Cocktail **(C)**: CD11c, CD14, CD19, CD94, CD123, FCER1α, TCRαβ, TCRγδ; Lineage Cocktail **(D)**: CD11c, CD14, CD19, CD94, CD123, FCER1α.

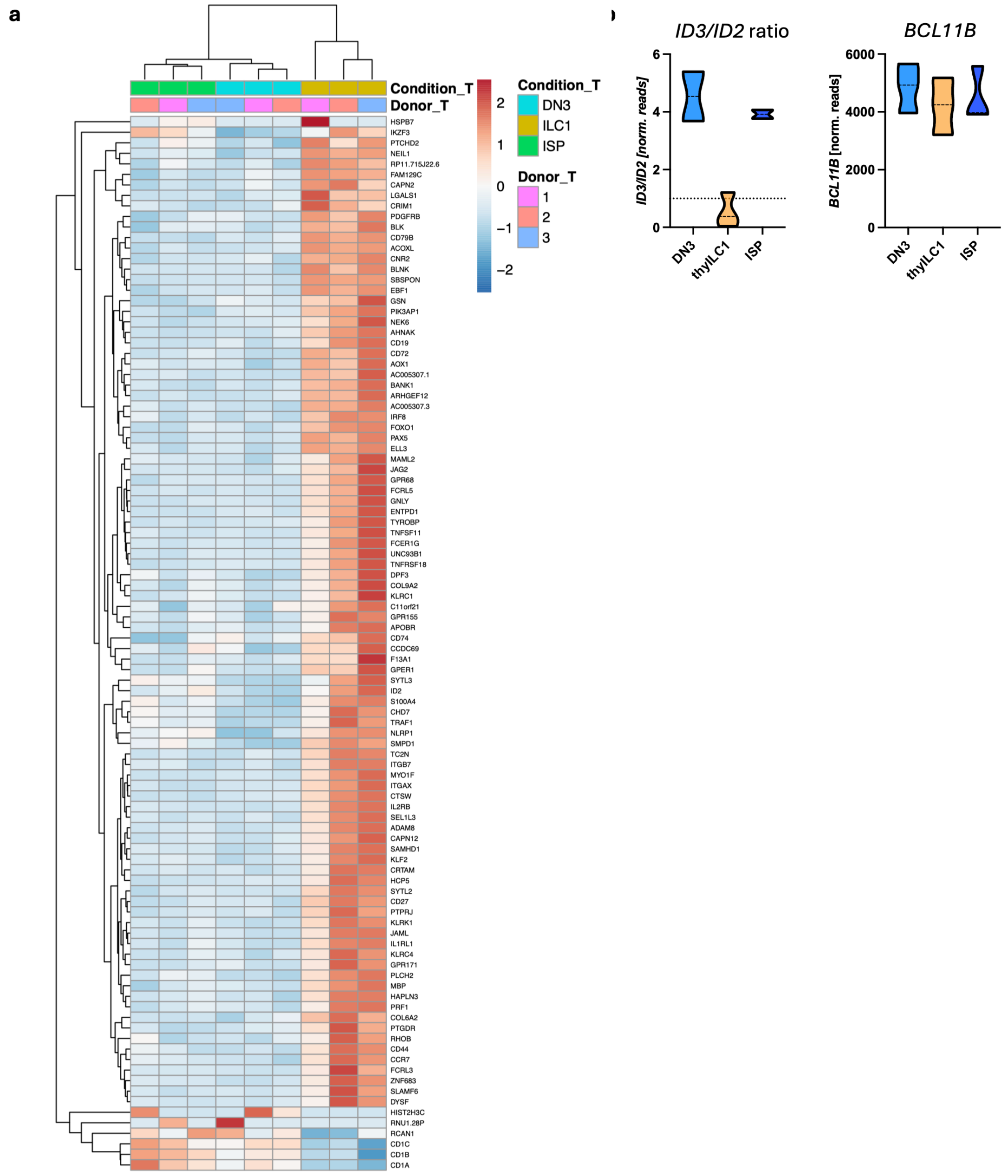

Supplementary Figure 5 | ThyILC1 show a unique transcriptional pattern compared to DN3s and ISPs in bulk-RNAseq.

Double Negative 3s (DN3s), thymic Innate Lymphoid Cells 1 (thyILC1s) and Immature Single Positives (ISPs) (10.000 – 20.000 cells each) were FACS sorted (suppl. Figure 1 (A) and (B)) for subsequent bulk-RNAseq. The data was analyzed using the DESeq2 R package. The Heat map shows the top 100 differentially expressed genes between DN3s (blue) and thyILC1s (yellow) including ISPs (green) (n= 3) **(A)**. Violin plots showing the *ID3/ID2* ratio of normalized read counts (left hand side) and normalized read counts of BCL11B (right hand side, n=3) **(B)**.

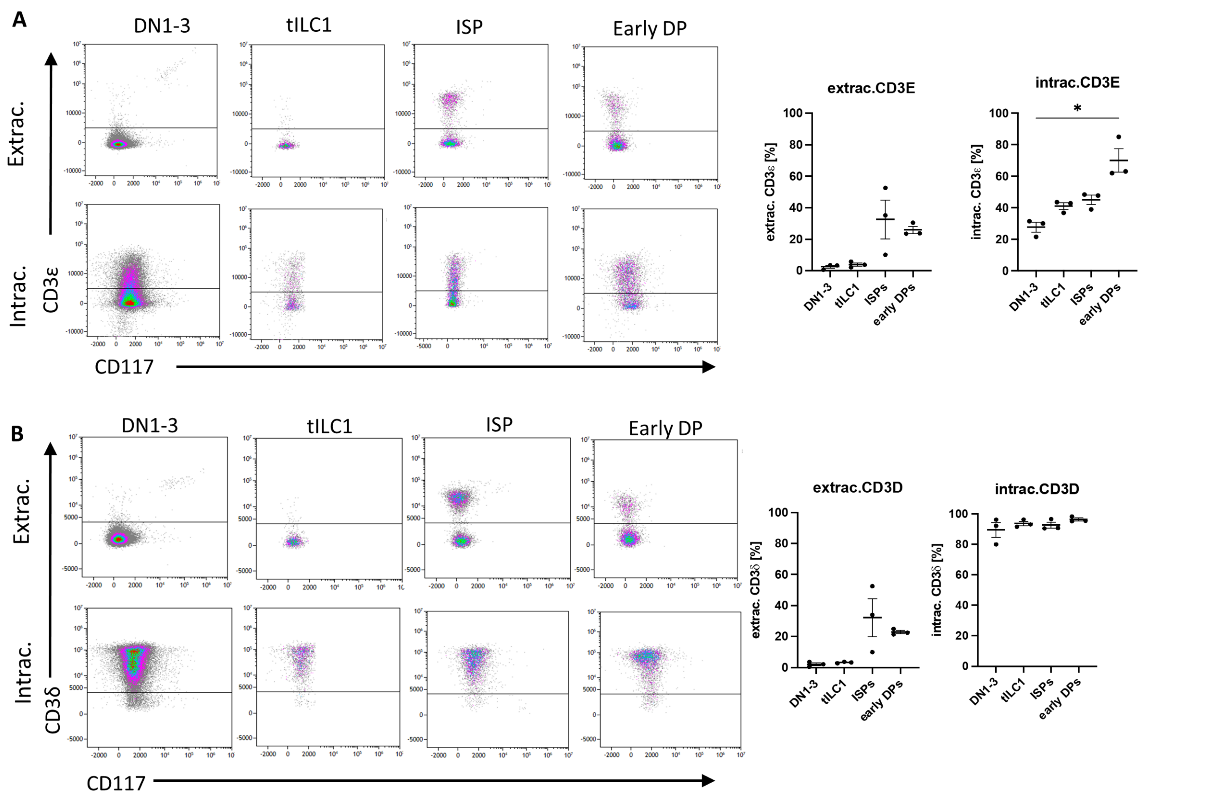

Supplementary Figure 6 | ThyILC1s show intracellular expression of CD3ε and CD3δ. Thymocytes isolated from postnatal thymi (PNT) from pediatric patients were analyzed for the expression of extra- and intracellular CD3ε and CD3δ chains. Representative dot plots and quantification in a bar chart for extracellular (top) and intracellular (bottom) expression of the CD3ε **(A)** and CD3δ **(B)** chain from (DN1-3, thyILC1s, ISPs and early Double Positives (DPs) (defined as shown in Figure 3 **(A)**) ($n = 3$). The height of the bars represents the mean ± SEM. The levels of significance were calculated with a non-parametric ANOVA (Kruskal-Wallis Test) with multiple comparison post-test between all populations. Levels of significance were indicated as * $p < 0.05$.

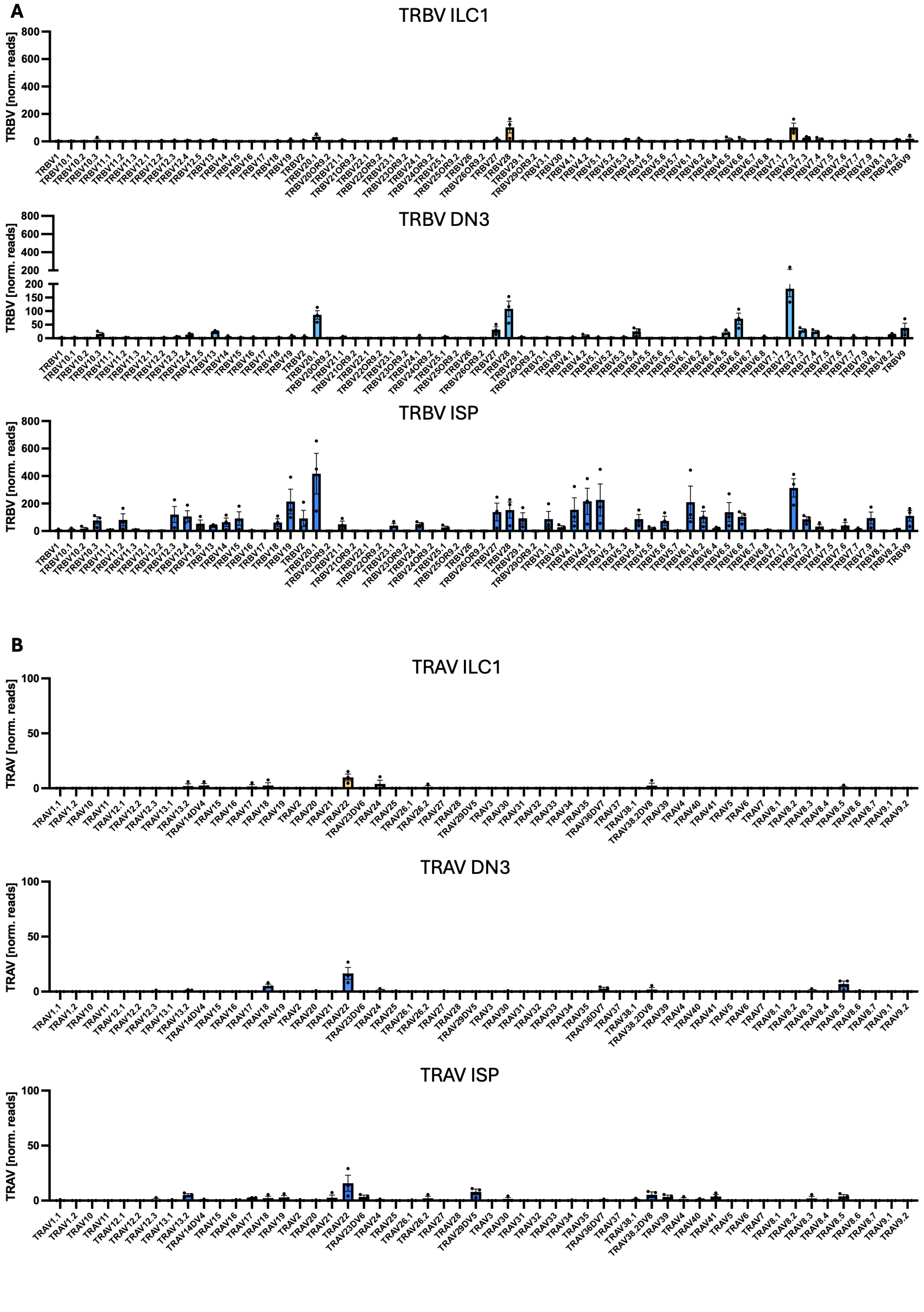

Supplementary Figure 7 | Individual *TRBV* and *TRAV* gene counts for thyILC1s, DN3s, and ISPs.

Normalized read counts of the individual *TRBV* **(A)** and *TRAV* **(B)** genes for thyILC1s (yellow bars), DN3s (light blue) and ISPs (dark blue) extracted from the bulk-RNAseq data set (n = 3). The height of the bars represents the mean ± SEM.

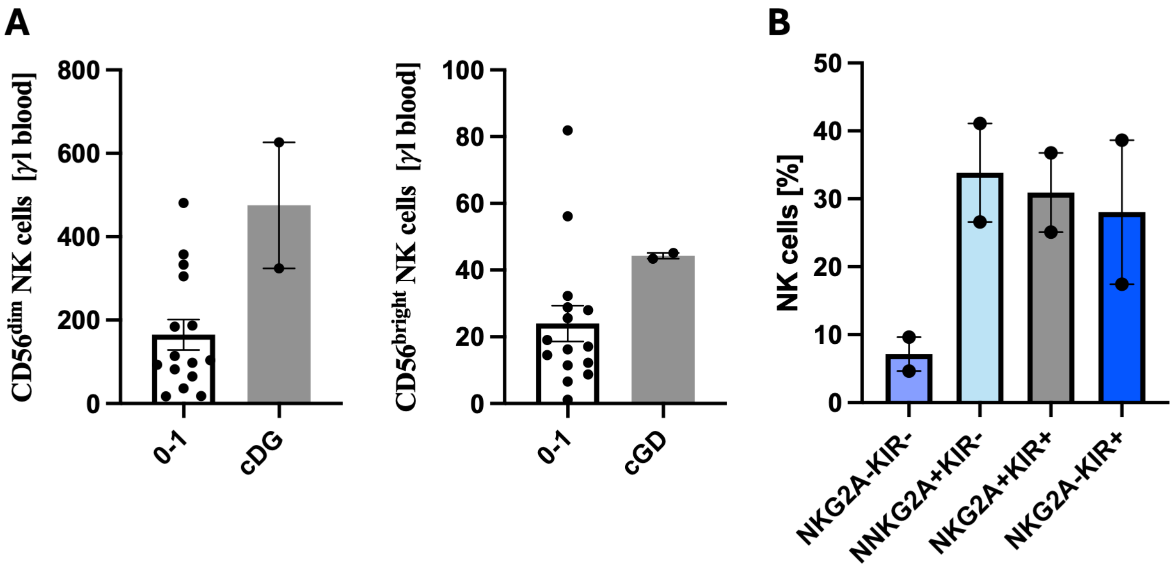

Supplementary Figure 8 | NK cell counts, KIR and NKG2A expression in patients with a 22q11.2del

Specifics for the patient samples please look at Figure 6. Bar graphs showing total cell counts (per µL blood) for CD56^{dim} and CD56^{bright} NK cells from 22q11.2del with congenital athymia, also known as complete DiGeorge syndrome (n = 2) in comparison to age-matched healthy controls (n=15). **(e)** Bar graphs showing the frequencies of NK cell subsets based on NKG2A and KIR expression in patients with 22q11.2del with congenital athymia (n=2). The height of the bars represents the mean ± SEM. Due to the small sample size, no statistical tests could be calculated with the DiGeorge patients.

Supplementary Table 1 – BD® AbSeq Immune Discovery Panel Specificities

Specificity	Clone	Oligo ID
CD3	UCHT1	AHS0231
CD4	SK3	AHS0032
CD8	SK1	AHS0228
CD11c	B-Ly6	AHS0056
CD14	MPHIP9	AHS0037
CD16	3G8	AHS0053
CD19	SJ25C1	AHS0030
CD25	2A3	AHS0026
CD27	M-T271	AHS0025
CD28	L293	AHS0138
CD45RA	HI100	AHS0009
CD56	NCAM16	AHS0019
CD62L	DREG-56	AHS0049
CD127	HIL-7R-M21	AHS0028
CD134	ACT35	AHS0013
CD137	4B4-1	AHS0003
CD161	HP-3G10	AHS0205
CD183 (CXCR3)	1C6/CXCR3	AHS0031
CD185 (CXCR5)	RF8B2	AHS0039
CD186 (CXCR6)	13B 1E5	AHS0148
CD196 (CCR6)	11A9	AHS0034
CD197 (CCR7)	2-L1-A	AHS0273
CD272	J168-540	AHS0052
CD278	DX29	AHS0012
CD279	EH12.1	AHS0014
CD357 (GITR)	V27-580	AHS0104
CD366 (TIM-3)	7D3	AHS0016
HLA-DR	G46-6	AHS0035
IgD	IA6-2	AHS0058

Supplementary Table 2 – used antibodies

Antigen	Conjugate	Clone	Dilution	Company
CD1a	FITC<br>AF700	HHII49<br>HHII49	1:50<br>1:50	BioLegend, California<br>BioLegend, California
CD3	Biotin<br>BV785	OKT3<br>OKT3	1:40<br>1:40	BioLegend, California<br>BioLegend, California
CD3	FITC<br>BV605<br>APC-Cy7	UCTH1<br>UCTH1<br>UCTH1	1:50<br>1:50<br>1:50	BioLegend, California<br>BioLegend, California<br>BioLegend, California
CD3 δ	PE	7D6	1:50	Invitrogen
CD4	BV605<br>APC	OKT4<br>OKT4	1:100<br>1:50	BioLegend, California<br>BioLegend, California
CD5	APC-Cy 7<br>BV605	L17F12<br>L17F12	1:50<br>1:50	BioLegend, California<br>BioLegend, California
CD8	BV510<br>APC-Cy7<br>AF700	RPA-T8<br>RPA-T8<br>RPA-T8	1:50<br>1:50<br>1:50	BioLegend, California<br>BioLegend, California<br>BioLegend, California
CD11c	FITC	3.9	1:50	BioLegend, California
CD14	FITC	HCD14	1:50	BioLegend, California
CD16	BV605	3G8	1:40	BioLegend, California
CD19	FITC	HIB19	1:50	BioLegend, California
CD34	FITC<br>PE	582<br>582	1:100<br>1:50	BioLegend, California<br>BioLegend, California
CD56	BV650	HCD56	1:50	BioLegend, California
CD94	PE-Cy7<br>APC	DX22<br>DX22	1:100<br>1:100	BioLegend, California<br>BioLegend, California
CD107a	BV510	H4A3	1:50	BioLegend, California
CD117 (c-Kit)	PE-Cy7<br>BV421	104D2<br>104D2	1:100<br>1:100	BioLegend, California<br>BioLegend, California
CD123	FITC	6H6	1:50	BioLegend, California
CD127	PE-Cy5	R34.34	1:40	Beckmann Coulter
CD158 a (KIR2DL1)	APC	143211	1:12,5	R&D,
CD158 a, h, g (KIR2DL1/ S1/ S3/ S5)	FITC<br>PE	HPMA4<br>HPMA4	1:100<br>1:100	BioLegend, California<br>BioLegend, California

CD158 b1, b2, j (KIR2DL2/ L3/ S2)	PE	DX27	1:100	BioLegend, California
CD158 b1, b2, j (KIR2DL2/ L3/ S2)	PE-Cy5	GI183	1:50	Beckmann Coulter,
CD158 b2 (KIR2DL3)	FITC	180701	1:50	R&D
CD158 e1 (KIR3DL1)	PE<br>AF700	DX9<br>DX9	1:100<br>1:400	BioLegend, California<br>BioLegend, California
CD159a (NKG2A)	APC	Z199	1:100	Beckmann Coulter,
CD159c (NKG2C)	AF700	134522	1:50	R&D
CD161	AF700	HP-3G10	1:25	BioLegend, California
CD200R1	PE-Cy7	OX-108	1:50	BioLegend, California
CD235a	FITC	HI264	1:50	BioLegend, California
CD294 (CRTH2)	PE-Dazzle<br>BV510	BM16<br>BM16	1:40<br>1:40	BioLegend, California<br>BioLegend, California
CD335 (NKp46)	BV510	9E2	1:50	BioLegend, California
CD336 (NKp44)	APC-Cy7	P44-8	1:50	BioLegend, California
FcεRIα	FITC	AER-37	1:50	BioLegend, California
IFNγ	AF700	B27	1:50	BioLegend, California
NKp80	APC	5D12	1:50	BioLegend, California
TCRαβ	FITC	IP26	1:50	BioLegend, California
TCR C β 1	APC	JOVI.1	1:50	BioLegend, California
TCRγδ	FITC<br>BV510	B1<br>B1	1:50<br>1:50	BioLegend, California<br>BioLegend, California
Isotype control IgG1	AF700	MOPC-21	1:50	Biolegend, California
